# Environmental Exposure to Per- and Polyfluorylalkyl Substances (PFASs) and Reproductive Outcomes in the General Population: A Systematic Review of Epidemiological Studies

**DOI:** 10.3390/ijerph21121615

**Published:** 2024-12-02

**Authors:** Alex Haimbaugh, Danielle N. Meyer, Mackenzie L. Connell, Jessica Blount-Pacheco, Dienye Tolofari, Gabrielle Gonzalez, Dayita Banerjee, John Norton, Carol J. Miller, Tracie R. Baker

**Affiliations:** 1 Department of Pharmacology, Wayne State University, Detroit, MI 48202, USA; a.haimbaugh@phhp.ufl.edu (A.H.); jessicarblount@gmail.com (J.B.-P.); 2 Department of Environmental and Global Health, University of Florida, Gainesville, FL 32611, USA; danielle.meyer@ufl.edu (D.N.M.); mconnell@ufl.edu (M.L.C.); ggonzalez6@ufl.edu (G.G.); banerj46@msu.edu (D.B.); 3 Great Lakes Water Authority, Detroit, MI 48226, USA; dienye.tolofari@glwater.org (D.T.); john.norton@glwater.org (J.N.); 4 Department of Civil and Environmental Engineering, Wayne State University, Detroit, MI 48202, USA; ab1421@wayne.edu

**Keywords:** PFAS, fertility, preterm birth, miscarriage, menopause, menstruation, PCOS, endometriosis, sperm, fetal growth

## Abstract

This Preferred Reporting Items for Systematic Reviews and Meta-Analyses (PRISMA) systematic review synthesized effects of background levels of per- and polyfluorylalkyl substance (PFAS) levels on reproductive health outcomes in the general public: fertility, preterm birth, miscarriage, ovarian health, menstruation, menopause, sperm health, and in utero fetal growth. The inclusion criteria included original research (or primary) studies, human subjects, and investigation of outcomes of interest following non-occupational exposures. It drew from four databases (Web of Science, PubMed, Embase and Health and Environmental Research Online (HERO)) using a standardized search string for all studies published between 1 January 2017 and 13 April 2022. Risk of bias was assessed by two independent reviewers. Data were extracted and reviewed by multiple reviewers. Each study was summarized under its outcome in terms of methodology and results and placed in context, with recommendations for future research. Of 1712 records identified, 30 were eligible, with a total of 27,901 participants (33 datasets, as three studies included multiple outcomes). There was no effect of background levels of PFAS on fertility. There were weakly to moderately increased odds of preterm birth with higher perfluorooctane sulfonic acid (PFOS) levels; the same for miscarriage with perfluorooctanoic acid (PFOA) levels. There was limited yet suggestive evidence for a link between PFAS and early menopause and primary ovarian insufficiency; menstrual cycle characteristics were inconsistent. PFAS moderately increased odds of PCOS- and endometriosis-related infertility, respectively. Sperm motility and DNA health were moderately impaired by multiple PFAS. Fetal growth findings were inconsistent. This review may be used to inform forthcoming drinking water standards and policy initiatives regarding PFAS compounds and drinking water. Future reviews would benefit from more recent studies. Larger studies in these areas are warranted. Future studies should plan large cohorts and open access data availability to capture small effects and serve the public. Funding: Great Lakes Water Authority (Detroit, MI), the Erb Family Foundation through Healthy Urban Waters at Wayne State University (Detroit, MI), and Wayne State University CLEAR Superfund Research (NIH P42ES030991).

## 1. Introduction

The objective of this systematic review is to synthesize recent results from quality studies on background levels of per- and polyfluorylalkyl substances (PFAS) and reproductive outcomes: fertility, preterm birth, miscarriage, menstruation and menopause, ovarian health, sperm health, and fetal growth. We present a rigorous, reproducible and informative review to be used by community organizations, government, industry, academia, and society.

It is a public health imperative to study any possible effects of xenobiotics on reproduction. The developmental origins of health and disease (DOHaD) framework theorizes the perinatal environment significantly impacts future health, and later health effects can be traced back to events in certain critical windows during growth [1]. Issues during pregnancy and development can extend to future generations, underscoring the requirement to study these early life effects now. Placental health is essential for protecting a fetus that has not fully developed its own xenobiotic detoxification system. Gestation is a critical window of development during which the timing of exposures can differentially actuate long-term effects. Despite limited studies on PFAS and the placenta, it is known that environmental contaminants in the placenta can contribute to adverse health effects [2,3,4] as PFAS readily cross the placental barrier [5], accumulating in fetal organs [6]. However, the long-term effects of maternal and placental PFAS exposure are largely unknown.

PFAS are a class of persistent chemicals characterized by a fluorinated carbon tail attached to a functional group. PFAS are ubiquitous, mobile and persistent in the environment and human blood across the world [7,8,9,10,11]. The carbon–fluorine bonds are the strongest in organic chemistry [12], rendering them virtually indestructible: a valuable trait for industrial uses and consumer products. PFAS have long been used for waterproofing and nonstick coatings, and more recently for electroplating and firefighting gear and foams [13]. One type of PFAS, perfluorooctanoic acid (PFOA), has been manufactured in the US since the 1940s [14] and has since been tied to numerous health effects in humans including reproductive issues [15]. The closely related perfluorooctane sulfonic acid (PFOS) has demonstrated negative health effects as well. The USA started regularly blood testing a representative sample of the public for PFAS in 1999 [16], finding PFOS in 99.9%, and three other PFAS (PFOA, perfluorohexane sulfonic acid (PFHxS), and perfluorononanoic acid (PFNA)) in >98% of blood samples from 2002 to 2003. Detection rates remain the same for the latest National Health and Nutrition Examination Survey (NHANES) data from 2017 to 2018 [17]. The half-life of PFOA and PFOS in blood is 3.5 and 4.8 years, respectively [18].

Adequate publicly available human data remain insufficient to thoroughly assess and regulate the vast majority of the thousands of chemicals that fall under the broad category of PFAS [3]. Other lesser-known PFAS continue to rise [19,20]. Predictions of alternative chemistries with lower toxicity have proven, at times, to be overly optimistic, earning some compounds the nickname “regrettable substitution.” For example, the main PFOS alternative in China, F-53B (also known as 6:2 Cl-PFESA), has the longest known PFAS half-life of 15.3 years [19,21]. The Environmental Protection Agency (EPA) has designated the chronic reference dose (RfD) for GenX (perfluoro-2-propoxypropanoic acid), the main PFOA alternative in the USA, as 3 ng/kg/d [14] compared to the current PFOA Rfd of 20 ng/kg/d [22]. Intermediate metabolites from fluorotelomer transformation “have been observed to be up to 10,000 times more toxic than [the parent PFAS]” [23]. Thus, rather than focusing on PFOA and PFOS in this review, we included every PFAS detected in ≥51% of each study’s samples above the LOD to capture both legacy and emerging PFAS.

The exact mechanisms of how PFAS exert their effects are unknown in most etiologies but are now understood as a class of endocrine-disrupting compounds (EDCs) in females and males. Pregnancy is a window of dramatic physiological changes affecting both mother and fetus, and both are especially sensitive to exposure during it. High levels of PFAS during pregnancy can cause gestational hypertension (GH) in the mother [24], which can have long-lasting effects on the mother and the child; GH is strongly associated with risk of subsequent cardiovascular disease in the mother [25] and increases the offspring’s risk of hypertension [26] and possible cardiac remodeling later in life [27]. Higher PFAS levels equated to shorter breastfeeding duration in five of the six included studies in a 2023 epidemiological review [28]. PFAS-associated altered breast development, shortened breastfeeding duration, and breast cancer in women are additionally reviewed in [29]. Out of 13 studies included between two systematic reviews, N-ethylperfluorooctane sulfonamidoacetic acid (EtFOSAA) [30], PFOA [31], and PFOS [30,32] were sometimes associated with increased time to pregnancy [33,34].

Male reproduction is relatively understudied, despite males being half the contribution to fertility and semen being an easily obtainable and non-invasive media. Previous reviews covered sperm concentration, count, motility, and morphology, and generally found PFAS unfavorably impacted these outcomes, though some studies found no associations [35,36,37]. Moreover, environmental stressors in sperm can have multi- or transgenerational influence on male infertility [38,39].

Epigenetics is recognized as a potential mechanism of transmitting health effects over multiple generations [40]. Periconceptual environmental conditions can cause epigenetic changes that persist even decades after the environment has changed for the former fetus [41]. PCOS’ heritability is thought to be genetic, epigenetic, and environmental [42]. A large retrospective cohort study found women who were born preterm were significantly more likely to deliver preterm babies of their own [43]. Regardless of mechanism, in utero exposures have long-term effects. For example, the exposure timing–outcome receipt of the teratogen thalidomide is so sensitive that the birth defect coloboma can be traced back to maternal use specifically during days 24–26 of gestation [44]. This concept is illustrated again in the lowered sperm quality of men previously exposed in utero following the dioxin plant accident in Seveso, Italy where their mothers lived [45], while male residents exposed as adults with fully intact reproductive systems were unaffected by the incident [46].

Tap water is a significant route of PFAS exposure, accounting for 2–34% of intake, with many regulatory agencies using 20% as a standard [47]. PFAS are present in the majority of tested community drinking water systems [48], which were not originally designed to filter out these chemicals. The half-life of PFOA in water is estimated at >92 years, and PFOS at >41 years [49,50,51]. USA drinking water treatment plants are chronically underfunded and can lack basic safety features [52]. An encouraging product of massive efforts by PFAS researchers, policy makers, and grassroots organizers is the 2021 Bipartisan Infrastructure Law (BIL), granting over USD 50 billion to improve water infrastructure, though the 2019–2039 cumulative investment gap is thought to be over USD 2 trillion [53]. USD 5 billion in the BIL is allocated for PFAS in drinking water through 2026 [54]; much more will be needed. In the absence of enforceable federal PFAS limits in drinking water, some states have begun passing legislation limiting the amount of allowable PFAS in drinking water to protect their constituents, despite the technological, political, and financial challenges this creates [55]. One utility of this systematic review is to inform decision makers (risk assessment managers, policymakers, local governments, etc.) in stewardship of allocating valuable, scarce, and competing water resources of the realistic risks posed by PFAS and the resulting importance of mitigating current PFAS intake stemming from drinking water.

This systematic review’s impetus is to inform the public and scientific community about reproductive health effects associated with background PFAS levels in the general population using a thorough and reproducible method of inquiry.

## 2. Materials and Methods

### 2.1. Eligibility Criteria

We assessed peer-reviewed published reports in the years of 2017–2022 in the English language. We excluded studies on any non-human model, infants, or children, occupational exposure or exposure through pollution of drinking water (high exposure groups), reviews, meta-analyses or abstracts; we also excluded studies with medium or higher risk of bias score (see Section 2.7). Other related reproductive outcomes were excluded due to existing extensive or recent reviews: gestational weight gain, gestational diabetes, gestational hypertension, preeclampsia, time to pregnancy, and hormones. All study types were permitted (cohort, case–control, etc.). Inclusion criteria covered community samples and those seeking assistive reproductive technologies (ARTs). In reports that contained included and excluded outcomes, we reviewed only the included outcomes. Reports were grouped into seven categories for analysis: fertility, preterm birth/gestational age at birth, miscarriage, menopause/menstruation, ovarian health, sperm health, and fetal growth.

### 2.2. Information Sources

The last date of each database search was 13 April 2022. Due to resource constraints, more recent studies are not included. We searched PubMed, Embase, Web of Science, and Health and Environmental Research Online (HERO).

### 2.3. Search Strategy

The search strategy included terms to capture all PFAS and outcomes of interest. Table 1 displays the advanced search terms for each database.

### 2.4. Selection Process

**Figure 1:**
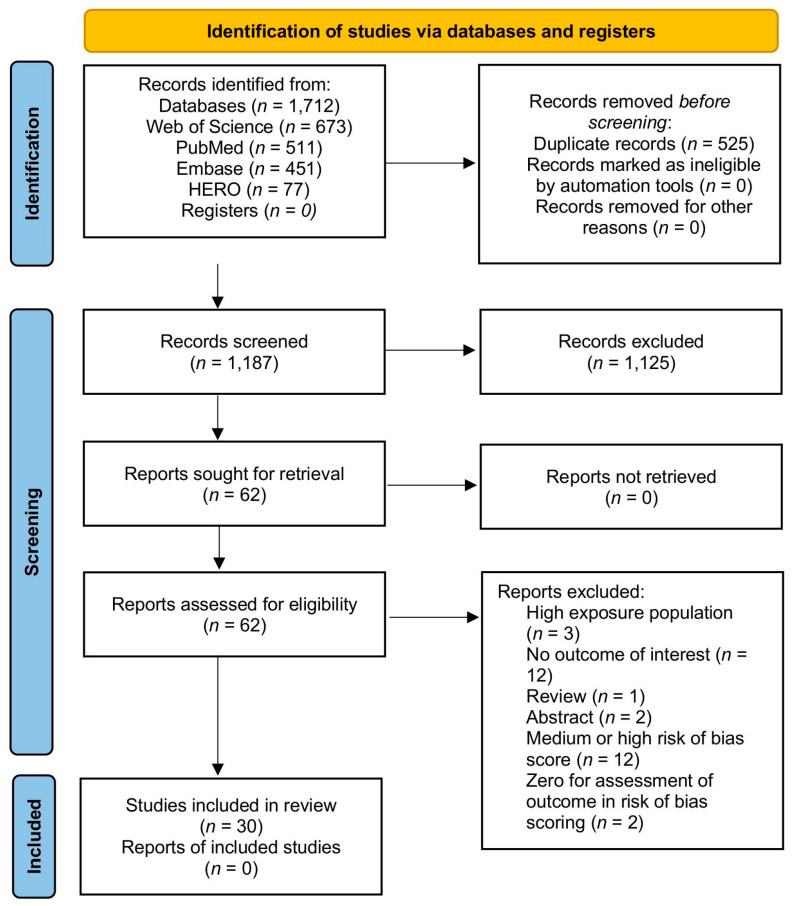
PRISMA flow diagram.

Results returned by the search terms were exported and loaded into SWIFT-Active Screener (Sciome, Triangle Park, NC, USA) [56]. At a minimum, this information included the title, authors, year, and abstract. SWIFT-Active Screener removed 525 duplicates before reviewer screening. Inclusion and exclusion criteria are listed above. At least two independent reviewers screened each returned report and marked them as eligible or ineligible for further analysis. Conflicts between independent reviewers at this and all other stages were resolved through discussion or by a third reviewer. Titles, authors, and year were exported for eligible reports. The filtering process is summarized in the PRISMA flow diagram (Figure 1) made using the template developed in [57].

### 2.5. Data Collection Process

One independent reviewer collected data from each report based on a common template that included the paper title, first author, PFAS, timepoint (if applicable), outcome, effect measure, covariates, unit of measurement, median concentration (or high/low concentrations for case–control studies), findings, media, country of study, and number of the table or figure from which the data were collected. This collection was reviewed by a second independent reviewer. If more data were collected by the second reviewer, the first reviewer reviewed the new collection. Any disagreements were resolved between the two reviewers. No automated tools or software were used.

### 2.6. Data Items

**Figure 2:**
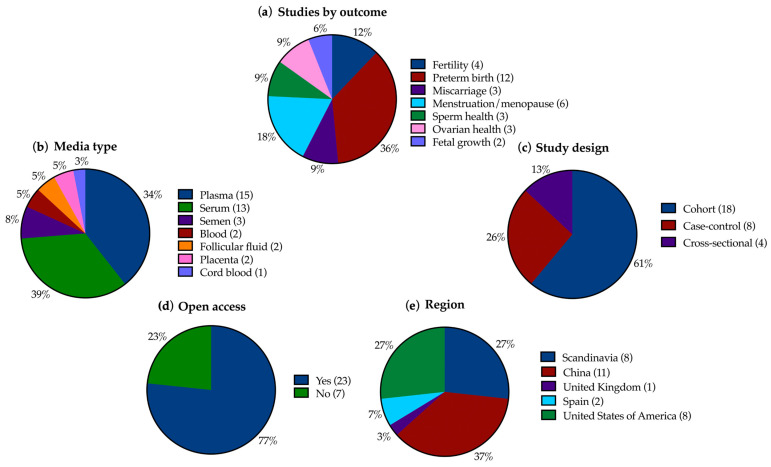
Pie charts depicting study characteristics. Percentages are rounded to nearest whole number and may not add up to 100%. (**a**) Number of studies included for each outcome. Some studies measured multiple outcomes. (**b**) Media type used in studies. Some studies used multiple media. (**c**) Study design type. (**d**) Open access status. (**e**) Region of studies. Scandinavia includes Sweden, Norway, Faroe Islands, and Denmark.

**Figure 3:**
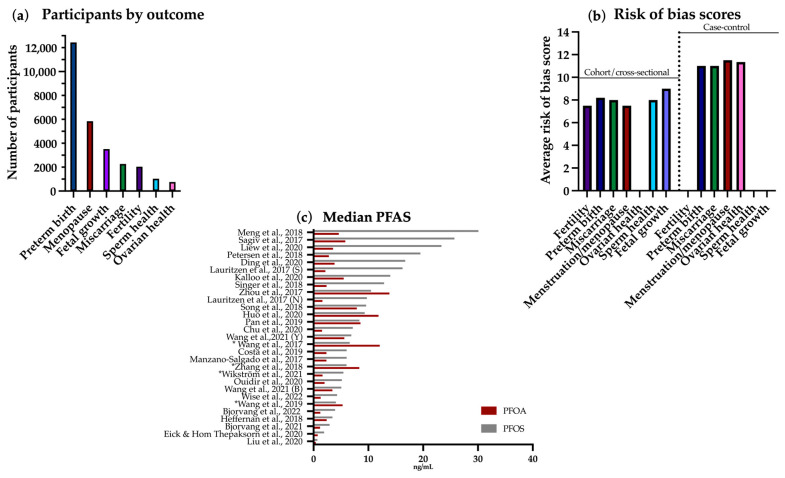
(**a**) Participants in each outcome. (**b**) Average risk of bias scores in each outcome stratified by cohort or cross-sectional studies (left) or case–control (right). Minimum points to be included in the review for cohort/cross-sectional was 7; maximum achievable was 10. Minimum points to be included in the review for case–control was 10; maximum achievable was 14. (**c**) Median PFAS levels (ng/mL) in each study in maternal/paternal blood, serum, or plasma. Levels reported from controls only in case–control studies. Multi-region studies denoted with (S) for Sweden, (N) for Norway, (Y) for Yantai, and (B) for Beijing. Meng et al., 2018 [58]. Sagiv et al., 2017 [59]. Liew et al., 2020 [60]. Petersen et al., 2018 [61]. Ding et al., 2020 [62]. Lauritzen et al., 2017 [63]. Kalloo et al., 2020 [64]. Singer et al., 2018 [65]. Zhou et al., 2017 [66]. Song et al., 2018 [67]. Huo et al., 2020 [68]. Pan et al., 2019 [69]. Chu et al., 2020 [70]. Wang et al., 2021 [71]. Wang et al., 2017 [72]. Costa et al., 2019 [73]. Manzano-Salgado et al., 2017 [74]. Zhang et al., 2018 [75]. Wikström et al., 2021 [76]. Ouidir et al., 2020 [77]. Wang et al., 2021 [71]. Wise et al., 2022 [78]. Wang et al., 2019 [79]. Bjorvang et al., 2022 [80]. Heffernan et al., 2018 [81]. Bjorvang et al., 2021 [82]. Eick & Hom Thepaksorn et al., 2020 [83]. Liu et al., 2020 [84]. Asterisk denotes the concentration of controls in case-control studies, if overall median is not reported.

We sought data for outcomes of fertility (female), sperm health, miscarriage, fetal growth, preterm birth/gestational age at birth, menopause/menstruation, and ovarian health (endometriosis and PCOS). The PECO statement is shown in Table 2. Any measure and time frame of these outcomes were sought. For fertility, this included infertility, fecundity, IVF outcomes, ovarian reserve, parity, time since last birth, live births, follicle counts, etc. Preterm birth/gestational age at birth were live births in <37 week gestation. Miscarriage is spontaneous abortion of a conceptus. Menopause/menstruation included onset of either, and menstrual cycle characteristics such as length, regularity, and severity. Ovarian health included PCOS, PCOS-related infertility, and endometriosis-related infertility. Sperm health included motility, morphology, concentration, volume, and DNA instability. Fetal growth included in utero fetal morphometrics (infant morphometrics and small-for-gestational-age were not covered, as these are ex utero measurements). Country of study, media type, open access status, study design, number of participants, and median PFAS levels in participants were also sought (Figure 2 and Figure 3c).

### 2.7. Study Risk of Bias Assessment

We adapted the Newcastle–Ottawa scale for our needs [85,86] (File S1). No automated tools were used. At least two independent reviewers rated each report according to the scale. Conflicts between independent reviewers were resolved through discussion or a third reviewer. Cohort or cross-sectional studies scoring less than 5 points out of 10 were considered a very high risk of bias; 5–6 points a medium risk of bias, 7–8 points a low risk of bias, and 9–10 points a very low risk of bias. Case–control studies scoring less than 5 points out of 10 were considered a very high risk of bias; 5–9 points a medium risk of bias, 10–12 points a low risk of bias, and 13–14 points a very low risk of bias. Supplementary Tables S1 and S2 show the scoring for all domains in each included report. Reports scoring medium or very high were not included in further analysis. Any studies receiving a 0 in the “assessment of outcome” domain were not included.

### 2.8. Effect Measures

All data were collected from reports, including any effect measure. We did not synthesize any data for meta-analysis on any study. For correlations, we considered r values of <0.20 as very weak, <0.40 as weak, <0.60 as moderate, <0.80 as strong, and 0.81–100 as very strong [87]. For the regression coefficient β, effect sizes between 0.10 and 0.29 were considered weak; 0.30–0.49 moderate; and 0.50 or greater were strong [88]. For odds ratios, effect sizes around 1.5 were weak; 2.5 moderate; 4 strong; and 10+ very strong [89]. Percent changes between 15 and 85 used the descriptors of small for a 7-point difference; medium for 18; large for 30; and very large for 45 [89].

### 2.9. Synthesis Methods

For studies where data were available in the main or Supplementary Text/Tables, forest plots were generated from aggregated data for either beta values and 95% CIs or odds ratios and 95% CIs. No meta-analysis was conducted.

### 2.10. Reporting Bias Assessment and Certainty Assessment

No formal reporting bias or certainty assessment was conducted.

## 3. Results and Discussion

### 3.1. Included Studies: Overview and Characteristics

From 1712 records identified, 30 were eligible for inclusion. Three records included multiple outcomes. Studies were mainly conducted in China, the USA, and Scandinavia; additionally in Spain and the UK. In total, 61% were cohort studies, 26% were case–control studies and 13% were cross-sectional. The records considered the following seven reproductive outcomes (with number of included papers and total participants, respectively, revealed within parenthesis): fertility (4, 2039); preterm birth (12, 12,442), miscarriage (3, 2270), ovarian health (3, 761), menstruation and menopause (6, 5845), fetal growth (2, 3514); and sperm health (3, 1030). A summary of study characteristics including average risk of bias score is presented in Figure 2 and Figure 3. If participant data were analyzed for more than one outcome, they were counted in both outcomes.

### 3.2. Fertility

Fertility is the ability to produce children. Fertility is arguably the overarching outcome of this review; all other endpoints are simply contributing factors to fertility. Infertility is defined as unsuccessful conception following 12 months of unprotected intercourse [90]. Most studies in this section come from fertility clinics, where fertility and pregnancy can be accurately measured by ultrasonography, histology, and measuring hormones such as follicle stimulating hormone (FSH) and human chorionic gonadotropin (hCG). In general, there are few female fertility studies that focus on PFAS. Earlier reviews did not find convincing links to PFAS and time to pregnancy (TTP) [33,91], though more recent reviews find longer TTP and reduced odds of pregnancy tied to PFOA and PFOS levels; PFNA and PFHxS have conflicting findings [29,34].

Our search identified four studies on fertility: two cohort studies in Sweden, a prospective cohort study in China, and a cross-sectional study in the USA (Table 3). Two studies collected samples in assisted reproduction therapy (ART) clinics, one during elective C-sections at a hospital, and one at a clinic. There was a wide variety in the specific endpoints measured, though all support fertility. The highest concentrations in all studies were of PFOS and PFOA. The publication from China [71] found a maximum of about 7 ng/mL PFOS, and 6 ng/mL PFOA. In the Swedish and USA papers [78,80,82], PFOS was around 3–4 ng/mL, and PFOA around 1 ng/mL at the highest.

Bjorvang et al. (2021) [82] leveraged minimally invasive access to reproductive organs during elective C-section of a cohort of 50 women at Karolinska University Hospital Huddinge in Stockholm and biopsied ovarian cortical tissue to study ovarian reserve through histology. They found that first trimester serum PFAS concentration in serum made no difference in unilaminar, atretic, growing, or healthy follicle densities. Furthermore, there was no association with infertility as measured by time to pregnancy (TTP) > 12 months, as revealed from electronic medical records.

Some reproductive outcomes require more invasive assessment. Bjorvang et al. (2022) [80] collected serum and follicular fluid at ovum pickup from a cohort of 185 women undergoing assisted reproductive technology (ART) at the Carl von Linné Clinic in Uppsala, which allowed them access to fertilization outcomes such as antral follicle count (AFC) via ultrasound, evidence-based/standardized morphological embryo scoring [92], ovarian sensitivity index (log((number of oocytes retrieved/total FSH dose) × 1000), clinical pregnancy (gestational sac presence at 6–7 weeks via ultrasound), and infertility as determined by a reproductive endocrinologist. They also used clinical records to track live births following fresh or frozen transfer.

In plasma and follicular fluid, median PFOS levels were 3.9 and 3.5 ng/mL, respectively. PFOS showed a significant positive association with higher AFC. AFC is one estimate of ovarian reserve; the higher the AFC, the greater the reserve. Ovarian reserve is an indirect estimate of a woman’s remaining follicles [93]. However, there were no significant associations between any other PFAS in either medium with ovarian sensitivity index. PFOS and PFNA in both media were significantly associated with lower odds of having at least one top-quality embryo, though importantly, average embryo score was unaffected. PFAS may be implicated in some granular ART outcomes such as AFC and top-quality embryos, but did not affect overall fertility. There were no associations between any PFAS in either medium with clinical pregnancy or live birth from any type of embryo transfer (fresh or frozen).

Wang et al. (2021) [71] conducted their prospective cohort study with 305 women in an ART setting as well, and examined clinical pregnancy failure (CPF) and hCG test-negative (hCG(−)). CPF was confirmed by the absence of gestational sac and fetal heartbeat at Week 6 of gestation via ultrasound following an implantation attempt. hCG(−) was defined as <10 IU of human chorionic gonadotropin 14 days after embryo transfer. They enrolled roughly 150 women from each of the two study sites: the inland city of Beijing and the seaside city of Yantai, which are approximately 450 miles apart. Serum was taken the morning of the day before the treatment cycle.

There were no similarities in significant findings between the study sites. In Beijing, PFDA levels were significantly higher in CPF cases than in controls (median 0.56 ng/mL in cases; 0.38 ng/mL in controls), and risk ratios increased with increasing concentrations and with quartiles (aRR = 2.28 (95% CI: 1.02, 5.11); Ptrend = 0.02). These trends were reversed in Yantai (median 0.51 ng/mL in cases; 0.61 ng/mL in controls; aRR = 0.45 (95% CI: 0.23, 0.85); insignificant Ptrend for quartiles).

As for other PFAS, in adjusted models using quartiles of PFAS concentrations, there were no significant monotonic dose responses in CPF. PFBA decreased the risk ratio for CPF in Yantai only in Q2 and Q3, though the overall trend was insignificant (Q2 aRR = 0.66 (95% CI: 0.46, 0.96); Q3 aRR = 0.70 (95% CI: 0.52, 0.96)); when not classified by quartiles, there was no relationship. PFBA levels in Yantai were 0.32 ng/mL in controls and 0.28 ng/mL in cases.

For hCG(−) test, PFDA significantly decreased RRs with increasing concentration in Yantai only (aRR = 0.50 (95% CI: 0.26, 0.98). This trend was reversed in Beijing, though insignificantly; however, PFDA significantly increased RRs across quartiles (Ptrend = 0.04). PFDA levels in Yantai were slightly higher in controls (0.59 ng/mL) than in cases (0.52 ng/mL); Beijing displayed the reverse pattern (0.38 ng/mL in controls; 0.56 ng/mL in cases). PFOA showed an inverted U-shape response in Yantai only, with significance in Q3 only (aRR = 1.72 (95% CI: 1.01, 2.91)). PFOA levels in Yantai were 5.3 ng/mL in controls and 5.9 ng/mL in cases. No other PFAS showed significant trends for hCG(−).

When data from both sites were combined, there were no significant findings for any PFAS, for either CPF or hCG(−).

There was no consistent evidence for PFAS exposure influencing these two reproductive outcomes. The study site heavily influenced results. PFAS profiles were different between the two study sites in terms of concentration and detection rate. There was no consistent evidence for PFAS exposure influencing these two reproductive outcomes.

Wise et al. (2022) [78] used cross-sectional analysis of 1499 Black women participating in the Study of Environment, Lifestyle, and Fibroids (SELF) cohort study in Detroit, MI. Plasma was collected at enrollment. Despite the study title, enrolled women had not been diagnosed with fibroids. Parity and time since last birth were measured by questionnaire. They found significantly lower mean PFAS concentrations associated with at least one birth for PFOA, PFHxS, PFNA, PFOS, and PFDA (median levels were 1.3, 0.6, 0.5, 4.3, and 0.2 ng/mL, respectively. The largest differences were with PFHxS (−34.7%) and PFOA (−33.1%). Further, women with more recent births (<2 years ago) had medium to large decreases in PFAS levels (PFOA (−33%), PFHxS (−29%), PFNA (−25%), PFOS (−18%)). It is well known that birth is an excretion route for PFAS, and thus statements on the meaning of these findings may be limited by reverse causation [29,94]. These results may simply reflect that PFHxS and PFOA are most easily excreted via birth. In fact, a recent meta-analysis of transplacental transfer efficiencies (TTEs) found PFHxS and PFOA to have higher TTEs than PFDA, PFNA, and PFOS [95]. One limitation is that although the women were not clinically diagnosed with fibroids at enrollment, ultrasound examinations revealed undiagnosed fibroids in 22% of participants in the original study [96]. It was unclear what percentage of fibroids were in Wise et al. (2022)’s subset [78].

Taken together, these four studies show no evidence that PFAS affects fertility. Overarching endpoints of live births, clinical pregnancy, and infertility were unaffected, and ovarian structure supporting fertility was not negatively affected. Four high-quality studies are not enough to draw final conclusions regarding the multi-faceted endpoint of fertility. More studies are needed in each of the specific sub-outcome areas (i.e., parity, TTP, clinical pregnancy or failure from ART, etc.). Studies from ART centers are an excellent place to map clinical outcomes onto biological mechanisms, as they have the advantage of a more controlled environment than at-home fertilization and matched pathology not common to most hospitals seeing patients for infertility. In planning future fertility cohorts, study design is crucial to account for the potential confounding of changing pharmacokinetics in pregnancy, perhaps by drawing blood before pregnancy occurs and throughout the course of the pregnancy. The current paucity of evidence for PFAS affecting fertility may be due to the relative lack of studies, as pointed out in [97] by the authors’ review of EDCs, noting that generally, higher EDC concentrations are associated with sub- or infertility. Subfertility, with its subtler etiology, is more difficult to detect associations with than infertility, and it is a promising future direction for large cohort studies.

### 3.3. Preterm Birth/Gestational Age at Birth

Premature infants incur on average 4–10× greater hospital costs than term (≥37 weeks) infants [98,99]. Very premature infants drive this disparity; however, even a birth 1–2 weeks before term costs on average about 4× more than a term birth [99]. Economics aside, premature birth is deeply associated with many different adverse health outcomes later in life [100]. Approximately 10% of USA births are premature [79], and the reasons for premature birth are unknown in many cases. The environment can play a role. It is known that cigarette smoke [101] and heavy drinking [102,103] increase risk of premature birth. Given the known influence of other environmental exposures, we reviewed the literature for evidence of the effect of PFAS on this outcome that is often idiopathic. Interestingly, one narrative review reported no association in five studies of a highly exposed population, and a positive association with PFOS in the general population [104]. Two previous meta-analyses covering preterm birth (PTB) concurred that PFOS slightly increased the risk for PTB (OR range 1.01–1.20) [105,106], while another found no effect of PFOS, but found PFHpS significantly reduced GAB [107]. Four additional studies are included presently. Overall, we found little to no evidence of an association between PFAS and gestational age at birth (GAB) or PTB in the present review, including when stratifying for infant sex. PTB is a dichotomous endpoint (delivery earlier or later than 37 weeks gestation, while GAB is continuous (days/weeks)); a reduction in GAB by a few days may not always translate to a PTB outcome.

We included endpoints from twelve studies that met our inclusion criteria on both gestational age at birth (GAB; 11 studies) and preterm birth (PTB; <37 weeks gestation; 7 studies) (Table 4). Most quantified GAB and/or PTB by medical record abstraction. Manzano-Salgado et al. (2017) [74] used self-reported last menstrual period. Sagiv et al. (2017) [59], Meng et al. (2018) [58], and Lauritzen et al. (2017) [63] used ultrasound scan. Ten were cohort studies and two [84,108] were case–control studies. Five were conducted in the USA [59,64,83,109,110], four in China [68,70,84,108], and one each in Spain [74], Norway [63], and Denmark [58]. Infant sex is a relevant modifier to consider, as it is thought that PFAS as an endocrine disruptor may have differential effects based on sex. In vitro studies have found PFAS can activate sex-steroid hormone receptors [111,112]. In fact, correlations between maternal steroid hormones and PFAS concentrations have been shown to vary with the sex of the infant being carried [113], and researchers using NHANES data found PFOS, PFOA, and PFDeA appear to increase free testosterone in reproductive-aged women [114]. At the same time, it has also been shown in vitro that current serum levels of PFOA do not affect free testosterone [115]. More research is needed in this realm, and to the paper’s credit in this section, all but two reported infant sex [68,84].

Lauritzen et al. (2017) [63] analyzed a prospective, multi-center case–cohort study (USA National Institute of Child Health and Human Development (NICHD) Scandinavian Successive Small-for-Gestational Age births study) of 424 women from the 1980s. Serum samples were collected between gestational Weeks 17 and 20. GAB (weeks) was determined by ultrasound scan at Week 17 of gestation. PFAS levels were higher in the 1980s than today, as evidenced by the median PFOS level of 16.4/9.74 ng/mL (Sweden/Norway). Contemporary USA median PFOS levels are 3 ng/mL [116]. PFOA levels were comparable to the NHANES 2017–2018 median of 1.32 ng/mL [116] (2.33/1.62; Sweden/Norway). Even with higher levels of these legacy PFAS, no significant associations were found with GAB (weeks) in either country, including when stratified by infant sex.

Kalloo et al. (2020) [64] also found relatively high PFOS (14 ng/mL median) and PFOA (5.5 ng/mL) concentrations in their prospective pregnancy and birth cohort (Health Outcomes and Measure of the Environment (HOME) Study) of 380 women in the early to mid-2000s. Serum samples were collected at 16 or 26 weeks of gestation. GAB (weeks) and PTB were abstracted from medical records. Similar to the findings in [63], they found no significant associations with maternal serum PFAS and GAB in any model, including per IQR increase and in clusters with other contaminants. There was no difference by infant sex.

Manzano-Salgado et al. (2017) [74] used a larger (1202 women) prospective birth cohort (Environment and Childhood—INfancia y Medio Ambiente (INMA)) from the mid-2000s and found no significant associations between first trimester maternal plasma PFAS and GAB (weeks) or PTB in any models (ORs or regression), including infant sex. GAB was calculated using self-reported last menstrual period. Median PFOS was 6.05 ng/mL.

Bangma et al. (2020) [109] used placental samples taken at birth from a small cross-sectional cohort of high-risk pregnancies (122 women; UNC Preterm Birth Biobank Study). This study was one of three receiving the lowest risk of bias score (9 out of 10) for this outcome. “High risk” for spontaneous PTB was defined as a prior PTB, short mid-trimester transvaginal cervical length, gestation of multiples, and antepartum hospital admission for threatened preterm labor. As such, the heterogeneity of the group was low; however, the mean GAB and prevalence of PTB was different from the average USA population. PTB and GAB (trichotomized, weeks) were abstracted from medical records. About half of the participants delivered PTBs (53.3%).

There was no significant association with GAB or PTB and the three PFAS detected in placentas (PFOS, PFHxS, PFHpS), even when stratified by infant sex. Median levels in placenta (ng/g) were as follows: PFOS (0.48), PFHxs (0.07), PFHpS (0.01). Interestingly, PFOA was only detected > RL in 27% of placental samples and thus was not analyzed further. PFOA has been widely observed to easily transfer to the placenta from maternal blood [95], and was found in placenta in [110] described below.

Hall et al. (2022) [110] used a small (120 women) prospective pregnancy cohort study (Healthy Pregnancy, Healthy Baby (HPHB)) of primarily of non-Hispanic Black women, and measured PFAS in placental samples taken at birth to examine associations with GAB (days; abstracted from medical records). Overall, there were no significant findings: none with the race of mother and none with the total infants. Median levels of PFOA and PFOS were 0.27 and 0.95 ng/g, respectively. However, in male infants only, the highest tertile of PFDA exposure was associated with an earlier birth of about 10 days (B = −10.4 (95% CI: −18.21, −2.68), *p* = 0.01). Median PFDA level was 0.2 ng/g; tertile levels were not reported. This finding is in contrast to the sex-dependent results of [70], which found PFOA and PFOS decreased GAB in females, though PFAS levels were much higher in that study and PFDA was not included. Both studies were small cohorts and found no difference when males and females were combined. Interestingly, this study did not detect PFHxS in placenta, though it passes to the placenta from blood [95] and was found in [109].

Chu et al. (2020) [70] used a small (372 women) birth cohort study (Guangzhou Birth Cohort) in a large Chinese city to examine relationships between PFOS, PFOA, and 6:2 Cl-PFESA and both GAB and PTB. Serum was collected <3 days post-delivery. GAB (weeks) and PTB were abstracted from medical records. In addition, 6:2 Cl-PFESA is the longtime PFOS alternative of China historically unique to the country [117]. Median PFOA in serum was 1.54 ng/mL; PFOS 7.15 ng/mL; 6:2 Cl-PFESA 2.41 ng/mL. This study was one of three receiving the lowest risk of bias score (9 out of 10) for this outcome.

Both PFOS and 6:2 Cl-PFESA were significantly associated with an approximately 2–3 day decrease in GAB per ln-ng/mL increase in concentration. Additionally, Q4 6:2 Cl-PFESA concentrations were monotonically associated with a 5-day decrease in GAB compared to Q3 (P_trend_ < 0.001), though all of Q4 were still term births (37.9 weeks). When GAB was broken down by infant sex, males were unaffected, and female GAB was negatively affected by 6:2 Cl-PFESA, PFOS, and PFOA; each PFAS brought down GAB by 3–4 days. The authors concede the sex difference may be a chance effect from a small cohort. Further, if this decrease in GAB manifested, it would likely still result in a term birth. Although GAB was not pragmatically affected, PTB was associated with increases in PFAS concentrations. Odds of PTB were greater per ln-ng/mL increase in concentrations of 6:2 Cl-PFESA (2.67-fold (1.73, 4.15)) and PFOS (2.03-fold (1.24, 3.32)), and in Q4 vs. Q1 (5.42 (1.70, 17.29)) and 4.99-fold (1.34, 18.56)), respectively). PFOA was not associated. Infant sex was not examined for PTB. Many other PFAS were detected in this study but analyses were not reported (PFBA, PFDA, PFDoA, PFHpA, PFHpS, PFHxS, PFNA, PFUdA), which indicates there were no significant findings.

Eick and Hom Thepaksorn et al. (2020) [83] used a slightly larger (506 women) diverse prospective birth cohort (Chemicals in our Bodies (CIOB)) study and measured serum PFAS from between 12 and 28 weeks of gestation. GAB (weeks) and PTB were abstracted from medical records. No association was found with PFAS tertiles and ORs of PTB. The authors note that overall, there were no consistent associations between PFAS and decreased GAB (weeks) either. In fact, an adjusted model found a link between highest tertile Me-PFOSA-AcOH and increased GAB in female infants only (B = 0.79, 95% CI: 0.15, 1.43), though Me-PFOSA-AcOH levels were quite low (0.05 ng/mL median). Median PFOA and PFOS levels were 0.76 and 1.93 ng/mL, respectively. There was no evidence of PFAS being associated with earlier birth in any case.

Huo et al. (2020) [68] used maternal plasma from a large perspective cohort study (2849 mother–infant pairs; Shanghai Birth Cohort) and abstracted GAB (weeks) and PTB from medical records. Plasma was taken during gestational Weeks 13–16. This study was one of three receiving the lowest risk of bias score (9 out of 10) for this outcome. There were no links to GAB, and overall, no associations were found in many measures of PTB (overall PTB, indicated PTB, late PTB, indicated late PTB, spontaneous PTB, spontaneous late PTB, length of gestation). Median PFOA and PFOS levels were 11.85 and 9.33 ng/mL, respectively. “Non-spontaneous” PTB was analyzed for infant sex (not overall) and PFHxS significantly increased the risks in female infants only (aOR = 2.56 (95% CI: 1.18, 5.53)). Non-spontaneous PTB was not clearly defined in the text but is assumed to mean any type of indicated birth. Reasons for clinically indicated PTB vary in pathophysiology and severity in both the mother and fetus [118,119]; this study defined it broadly as being due to “preeclampsia, fetal stress, placenta previa and other maternal, fetal or placenta indications”. Thus, the risk female infants have of PTB may be due to a variety of factors besides PFAS. PFHxS levels were on the higher end of the included studies, but not the highest (0.56 ng/mL [64]: 1.5 ng/mL (no association)).

Yang et al. (2022) [108] used cord serum taken at delivery from a small PTB case–control study nested within the Kashgar Birth Cohort Study (384 matched case–controls). PTB was clinically diagnosed as birth between 28 and 36 weeks. Controls were matched by infant’s sex, delivery date, maternal age, and maternal residence distance to a factory. The recruiting hospital was in an area of low population density where the land use was mainly agricultural. As such, PFAS concentrations were relatively low in both cases and controls (median total PFAS: 1.727, 1.396 ng/mL (controls, cases); PFOA: 0.46, 0.29; linear PFOS: 0.17, 0.13). A total of 92% of the area’s population are from the Uyghur ethnic minority, which constitutes 58% of the study population.

There was a significant decrease in mean concentration in cases compared to controls for about 50% of the PFAS studied, and no significant difference in total PFAS. However, in adjusted ORs per IQR increase in concentration, total, linear, and branched PFOSs were significantly positively associated with PTB (aOR range 1.11–1.44, *p* < 0.01), though when stratifying by potential modifiers, there was no significant association. Per-IQR increase in total and linear PFOSs were further examined in relation to infant sex. There was no connection with infant sex and PTB for any PFOS group. Total, linear, and sum m2-PFOS were significantly inversely associated with GAB (weeks) in cases only (B range −3.03 to −1.26); additionally, PFDoA and PFHpS followed this significant trend (B = −3.43 (−5.55, −1.32); −3.02 (−4.93, −1.11), respectively). Linear PFOS was associated with infant sex GAB (P_interaction_ = 0.02), with the beta value for females (−2.32) being stronger than for males (−0.58); however, the CIs for both sexes contained zero. Though GAB may be affected, overall, there was no evidence for this decrease in GAB translating to clinical PTB in this study, and in fact controls had higher levels of most PFAS than did cases.

Meng et al. (2018) [58] drew from multiple subsamples of the Danish National Birth Cohort to analyze a pooled sample of 2137–3535 mothers (3535 for PFOA and PFOS samples; 2137 for the other PFAS) in Denmark. Plasma was taken once in the first and once in the second trimester. Median PFOS and PFOA levels were 30.1 and 4.6 ng/mL, respectively. This study’s median PFOS level is the highest of any included in the review. A total of 97% percent of GAB (weeks) records were verified by ultrasound before Week 24 of gestation; 3% were dated to last menstrual period.

PFDA and PFOS were significantly associated with slightly elevated odds of PTB per doubling of exposure (aOR = 1.7 (1.2, 2.5); 1.5 (1.1, 2.2), respectively), but not when examined by quartiles. PTB was not stratified by infant sex. Various PFAS affected GAB; however, not by more than 2 weeks. Notably, PFOS was associated with decreased GAB in both per doubling models (B = −1.1 (−1.7, −0.4)) and non-monotonically in all quartiles (Q4 B = −1.5 (−2.6, −0.5)). When stratified for infant sex, PFNA was significantly associated with decreased GAB in male infants only (B = −1.9 (−2.8, −0.5)). Overall, PFOS was the most frequently associated PFAS, though effect sizes were quite small. The following study showed similar results of PFOS mildly associated with increased PTB and decreased GAB, and PFNA affecting GAB in male infants only.

Sagiv et al. (2017) [59] drew 1645 participants from the Project Viva birth cohort in 1999–2002 eastern Massachusetts, US. Maternal plasma was taken at a median of 9 weeks of gestation. Median levels of PFOS and PFOA were 25.7 and 5.8 ng/mL, respectively. Most (79%) of gestational length measures were confirmed by ultrasound at 16–20 weeks of gestation, the rest were dated to last menstrual period (make sure this is correct interpretation). GAB was a dichotomous variable in this study only (<37 weeks vs. ≥37 weeks).

PFOS significantly increased odds for PTB in all quartiles of exposure (Q4 aOR = 2.4 (1.3, 4.4)), though this did not show in a continuous IQR model. PTB was not stratified by sex. PFOS was significantly negatively associated with GAB in quartiles only (Q4 B = −0.37 (−0.65, −0.10)), not in continuous IQR models. Exact statistics were not reported for interactions with infant sex; however, per IQR increase in both PFOS and PFNA, GAB was significantly decreased for males only.

Liu et al. (2020) [84] chose demographically matched cases and controls nested in a prospective cohort study (144 cases, 375 controls) in Shanxi Province, China. The participants were all women of Han ethnicity. Plasma was taken between Weeks 4 and 22 of gestation, and PTB was recorded by health care staff.

There was no association between PFAS levels and overall PTB or first-trimester PTB. For the second-trimester PTB, there was a significant positive association with PFOS levels (aOR = 3.35 (1.39, 8.05), *p* < 0.01). The median PFOS concentration was 1.79 ng/mL. The authors note the relatively low PFAS concentrations compared with other cohorts in the country and worldwide; the median PFOA level was 0.79 ng/mL. Further, they assert that even with the PFOS finding, it is unlikely PFAS at this level appreciably influences PTB.

**Figure 4:**
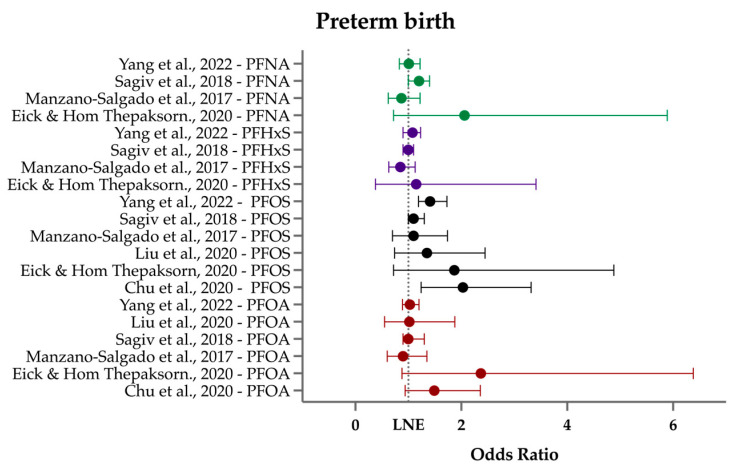
Forest plot of odds and risk ratios for preterm birth with increasing PFAS levels from four studies. LNE: line of no effect. PFNA: perfluorononanoic acid. PFHxS: perfluorohexane sulfonic acid. PFOS: perfluorooctane sulfonic acid. PFOA: perflurooctanoic acid. Yang et al., 2022 [108]. Sagiv et al., 2018 [59]. Manzano-Salgado et al., 2017 [74]. Eick & Hom Thepaksorn et al., 2020 [83]. Liu et al., 2020 [84]. Chu et al., 2020 [70].

In summary, most studies found no significant association between most PFASs and GAB or PTB. There were weakly to moderately increased odds of preterm birth with higher PFOS levels [59,70] and with 6:2 Cl-PFESA [70] (Figure 4). Though a few studies observed a decrease in GAB amounting to approximately 1–3 days [58,59,70,108,110], this difference is likely clinically irrelevant for PTB. While some studies observed sex-specific effects of PFAS on GAB, these findings were inconsistent. Future larger studies should include infant sex in their analysis, as it is still unknown whether there are sex-dependent differences in PFAS toxicity during gestation. Further, any associations with infant sex found in cohort studies should be examined mechanistically at the basic science level.

### 3.4. Miscarriage

Miscarriage (or spontaneous abortion) is a sentinel for subsequent obstetric difficulties and later in life health effects, including all-cause mortality [120,121]. Miscarriage, especially recurrent miscarriage, portends psychological issues for both members of a couple trying to conceive [122,123]. Studies on miscarriage and background levels of PFAS are sparse, and reviews even more so, despite certain environmental exposures having long been linked to miscarriage [124]. A study of highly PFOA-exposed women due to contaminated drinking water found no link to miscarriage, though interestingly, background levels of PFOS in the same population were associated with elevated odds of miscarriage (however, not significantly) [125]. There are two existing meta-analyses on background levels of PFAS and miscarriage. One included two studies and found a positive association with miscarriage and PFDA (pooled OR per 1-ng/mL increase: 1.87; 95% CI: 1.15, 3.03) [105]. The other used seven studies and found no significant associations with miscarriage [106]. Both meta-analyses included Liew et al. (2020) [60] (reviewed here) and other publications from before our inclusion period.

Three studies that examined miscarriage met all our criteria (Table 5). Two were Nordic case–control studies, one a Chinese prospective cohort study, and all used some component of blood. Two studied the first trimester, and one the second. Wang et al. (2021) [71] was conducted in an in vitro fertilization (IVF) setting, and as such, pregnancy was closely monitored: spontaneous clinical abortion was measured at 6 weeks. Wikström et al. (2021) [76] focused on the first trimester more broadly, and Liew et al. (2020) [60] on the second trimester. Five PFAS were detected in all studies: PFDA, PFHxS, PFNA, PFOA, and PFOS. PFOS was found at the highest concentration in all studies (range: 6.09 (Sweden)–24.55 ng/mL (Denmark) (cases)). PFOA ranges were tighter at 2 ng/mL (Sweden; cases)–3.97 ng/mL (China).

Following the timeline of gestation, we first discuss Wang et al. (2021) [71]. This prospective cohort study spanned two IVF sites in China (Beijing and Yantai) and included 305 women. Importantly, unlike the other studies, the populations were undergoing IVF, and as such, pregnancy and miscarriage (“spontaneous preclinical abortion”) were confirmed on defined days. To be considered a spontaneous preclinical abortion case, participants required a positive pregnancy test (hCG+) 14 d after embryo transfer and an ultrasonography-confirmed lack of gestational sac and heartbeat at 6 weeks. Serum was taken the day before IVF treatment began. Overall, there were no significant findings for the eight PFASs (PFBA, PFBS, PFDA, PFHxA, PFNA, PFOA, PFOS, PHFxS). PFHxA had no significant association when comparing median concentrations in cases vs. controls in adjusted or crude models at either site; however, when using quartiles of PFHxA in an adjusted model, there was a decreased relative risk (aRR = 0.12 (95% CI: 0.02, 0.93)) at the Beijing site in the third quartile only. Median levels of PFHxA in cases and controls were 0.21 and 0.17 ng/mL, respectively.

Wikström et al. (2021) [76] used ultrasound-confirmed first trimester miscarriages abstracted from clinical patient records in 78 cases and used 1449 controls from the Swedish Environmental Longitudinal Mother and child Asthma and allergy (SELMA) pregnancy cohort. They found PFOA was associated with a significantly increased OR of sporadic first trimester miscarriage in all three models (OR range: 1.38–2.66), which included crude OR not restricting to parous women. Median PFOA levels were 1.64 ng/mL in controls and 2 ng/mL in cases. No other PFASs (PFDA, PFHpA, PFHxS, PFNA, PFOS, PFUnDA) had any significant findings.

Liew et al. (2020) [60] was a retrospective case–control study utilizing the Danish National Birth Cohort, which enrolled women between 1996 and 2002. Plasma samples of 220 cases and 218 controls were collected at gestational Week 8, on average. Miscarriage data were obtained from the Danish National Hospital Discharge Register. Most PFASs (PFDA, PFHxS, PFNA, PFOS, PFOSA) showed no associations. Highest quartile PFOA and PFHpS were associated with significantly increased odds of miscarriage in some adjusted models (OR range PFOA: 1.9–2.4; PFHpS: 1.8–2.4). These held true when restricting to parous but not nulliparous women and when controlling for the additional factors of outcome of last pregnancy and time gap since last pregnancy; not when analyzing according to per-doubling increase in PFAS. Median PFOA levels were 3.56 ng/mL in controls and 3.96 ng/mL in cases. PFHpS concentrations were 0.36 ng/mL in controls and 0.39 ng/mL in cases. More recent data show both PFHpS and PFOA median serum levels are lower (0.2 ng/mL; 1.32 ng/mL, respectively) in the average American population in 2017 [116] than the population of Danish women in 1996–2002. PFHpS is an uncommon PFAS, and was not studied by NHANES in blood until 2017, where median levels were much lower (0.2 ng/mL) than PFOA (1.32 ng/mL) [116]. Even so, PFHpS has been shown to have a longer half-life in humans than PFOA or PFOS [126].

**Figure 5:**
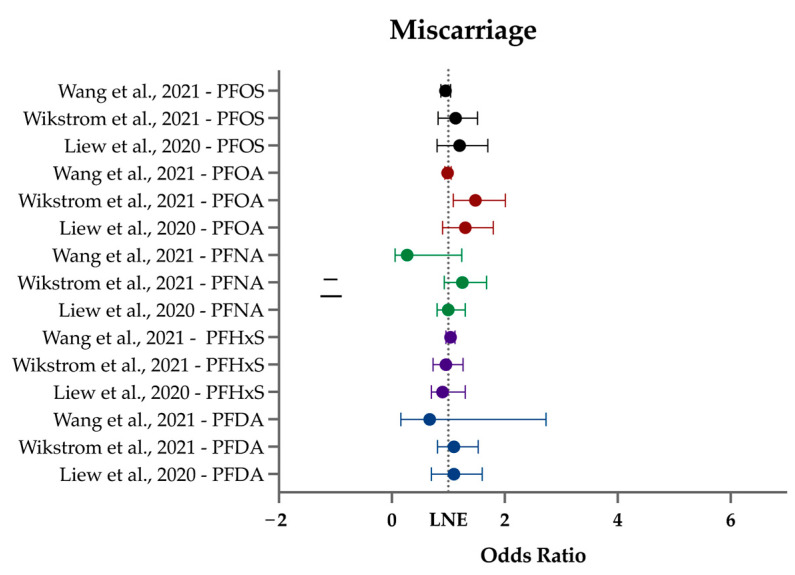
Forest plot of miscarriage odds and risk ratios with increasing PFAS levels from three studies. LNE: line of no effect. PFOS: perfluorooctane sulfonic acid. PFOA: perflurooctanoic acid. PFNA: perfluorononanoic acid. PFHxS: perfluorohexane sulfonic acid. PFDA: perfluorodecanoic acid. Wang et al. (2021) results are from Beijing and Yantai sites combined. Wang et al., 2021 [71]. Wikström et al., 2021 [76]. Liew et al., 2020 [60].

In sum, there is weak or no evidence for a relationship between most PFAS and miscarriage (Figure 5). In two of the three studies, PFOA was associated with weakly increased odds of miscarriage, especially in parous women. PFHpS was only included in one study and mirrored the outcomes of PFOA. The inclusion of this lesser-known PFAS and observed findings once again indicate the importance of including multiple PFAS in epidemiology studies, beyond PFOA and PFOS. We additionally recommend forthcoming research on miscarriage following the guidelines established by the European Society of Human Reproduction and Embryology to use consistent nomenclature in early pregnancy loss literature [127].

### 3.5. Menopause and Menstruation

Without menstruation, ovulation and pregnancy cannot result. Some EDCs can affect menstruation [128,129], ovulation [130,131], and ultimately fertility [132]. PFAS are increasingly viewed as EDCs; thus, all aspects of menstrual function must be studied. Menstrual bleeding is an important route of elimination for PFAS [133], accounting for 30% of the PFOS concentration discrepancy in men versus women [134]. Menopause is thus the cessation of a route of elimination, allowing compounds to more easily accumulate. Earlier or later menstruation or menopause affects the window of fertility in women. With the societal shift towards pregnancies occurring later in life [135], menopausal timing becomes more salient.

An existing systematic review of early menarche and PFAS [136] featured three studies on ambient exposure, none included here (two before inclusion period, one did not pass risk of bias assessment), and found inconsistent results. A recent narrative review [137] found no or conflicting associations with menarcheal timing, while another found insufficient evidence [138]. A comprehensive review of various ovarian health effects [139] found a wide body of associations with PFAS and cycle characteristics and menopausal timing; however, the authors concluded there is insufficient evidence at this time to draw causal relationships. We echo these reviews’ emphasis on the importance for more research into these health outcomes, as environmental exposures are an understudied potential contributor to them.

Five studies on menstruation and one on menopause met our inclusion criteria (Table 6). Two were case–control studies (UK [81], China [75]), and the rest were cohort studies (two in the US, one in Norway, and one in China). PFOA levels ranged from 1.3 ng/mL (USA) to 13.5 ng/mL (China); PFOS from 3.46 (UK) to 16.9 ng/mL (USA). PFOA was found at higher levels than PFOS in the Chinese studies; the reverse was true for all others.

Four additional studies (one on menopause, three on menstruation) failed the risk of bias assessment for receiving a zero in the assessment of outcome domain. Menstruation and menopause characteristics are difficult to track when relying on memory. Using self-recall alone was the reason these studies did not pass the assessment. Future studies could provide menstrual tracking supplies to prospective participants before menarche or base findings solely on menstrual cycles tracked in detail during the study period in girls or women who have already begun menstruation. More rigorous data collection will lead to more robust results on this outcome.

#### 3.5.1. Menopause

Ding et al. (2020) [62] used a longitudinal cohort of 1120 multiethnic premenopausal USA women aged 45–56 (Study of Women’s Health Across the Nation Multi-Pollutant Study (SWAN-MPS) cohort) to understand PFAS’ effect on menopausal timing. The SWAN study began in 1996, and the MPS portion in 2016. Researchers used repository serum samples taken at an earlier follow-up visit (1999–2000) and, through annual interviews, measured natural incident menopause, defined as 12 months of amenorrhea without any other cause. Unlike in most studies, they teased out linear and branched isomers of PFOA and PFOS. Linear PFOA (n-PFOA) and PFOS (n-PFOS) were detected in 99.9–100% of samples (median n-PFOA 4 ng/mL; n-PFOS 16.9 ng/mL), branched PFOA (Sb-PFOA) was detected in 18.2% of samples, and branched PFOS (Sm-PFOS) was detected in 99.9%.

Individually, increasing tertiles of n-PFOA, n-PFOS, and Sm-PFOS were all significantly associated with increased hazard ratios (HR approx. 1.30; 95% CI ranges 1.03–1.50) for earlier menopause for a predicted median difference of 0.9–1.1 years. This predicted difference increases to 2.0 years when examining the “high” total PFAS mixture group versus “low” total PFAS mixture group (HR = 1.63 (95% CI: 1.08, 2.45)). Measures reporting total PFAS are more realistic than individual PFAS alone, as we are always exposed to many at once.

The paper further breaks down HRs by a few minority groups (White, Black, Chinese, Japanese). This is important as different minority groups may have differing exposure to PFAS [140,141]. It finds only PFNA significantly associated with an increased HR (per doubling increase in concentration) in only White women (HR = 1.33 (95% CI: 1.13, 1.56)).

This study is evidence supporting the need for more research to understand a potential connection between menopause and ambient PFAS exposure. An earlier study using NHANES data [142] found an association with PFHxS, but none with PFOS, PFOA, or PFNA for early menopause. These studies both rightly warn about controlling for reverse causation in findings for menopause, as PFAS concentrations are higher after menopause due to an eliminated excretion route [134,141]. On the other hand, high exposure to PFOA, such as in the C8 Health Study, has been associated with earlier menopause [143]. Earlier menopause could prove especially relevant as more women plan to start or continue families later in life [144,145].

#### 3.5.2. Menstruation

While somewhat similar to menopause, primary ovarian insufficiency occurs when the ovaries stop working properly before the age of 40 [146]. Infertility can result. Diagnosis for POI includes irregular periods and consistently elevated FSH levels [147]. In most cases, POI is idiopathic, though environmental exposures including PFAS can play a part [148]. The case–control study of Zhang et al. (2018) [75] recruited 120 POI cases and 120 matched controls 20–40 years old during a hospital visit in China (First Affiliated Hospital of Nanjing Medical University) for irregular menstruation or amenorrhea. POI was defined as an elevated FSH level (>25 IU/L) on two occasions > 4 weeks apart and oligo/amenorrhea for at least 4 months. Menstrual history was collected at enrollment via interview, and plasma samples were taken. FSH levels were measured in this sample. It is unclear exactly where/when the requisite high FSH on another occasion was measured, though they state gynecological examination information was obtained from medical records; this may have included FSH testing. They found that PFHxS, PFOA, and PFOS levels were significantly positively associated with increased odds of POI in all models (aOR: 6.63, 3.80, 2.81, respectively; *p* < 0.01). Five other PFAS studied (PFBS, PFDeA, PFDoA, PFHpA, PFUA) had no associations. Median PFHxS levels were 0.29 ng/mL in controls and 0.38 ng/mL in cases; PFOA 8.35 in controls and 11.1 ng/mL in cases; PFOS 6.02 ng/mL in controls and 8.18 ng/mL in cases.

All other studies examined at least one aspect of menstrual cycle regularity or length. Zhou et al. (2017) [66] studied 950 women from the Shanghai Birth Cohort Study to produce odds ratios for PFAS and four aspects of menstruation: irregular cycle (cycle variations of >7 days between cycles), long cycles (>35 days), menorrhagia (heavy bleeding), or hypomenorrhea (abnormally low bleeding). Questionnaires were administered and blood samples drawn at enrollment. Median PFAS levels were 1.36, 13.84 (the highest median PFOA level of any study included in this review), and 10.49 ng/mL, respectively.

No significant associations were found for PFBS, PFDeA, PFDoA, PFHpS, PFOSA, or PFUA with any outcome. In general, the evidence for positive associations between four PFAS (PFHxS, PFNA, PFOA, PFOS) and irregular or long cycle was weak. Though ORs were statistically significant for these two outcomes, confidence intervals were very close to including one. Evidence was also weak for menorrhagia: crude and adjusted models show a significant negative association between all four PFAS levels and menorrhagia (aOR range: 0.14–0.57). In other words, odds decrease at least 43% for menorrhagia with increasing PFAS quartiles. No associations were found for hypomenorrhea.

Wise et al. (2022) [78] had similar findings for bleed intensity (light, medium, heavy; data collected via questionnaire) in their USA cohort study of 1499 women. Plasma sample questionnaires were obtained at enrollment. Nearly every PFAS studied (except MeFOSAA) had an inverse association with medium or heavy bleeding as compared to light bleeders (~ −10–20% difference); this includes PFHxS, PFNA, PFOA, PFOS as in [66], and additionally PFDA and PFUnDA. Median levels for these PFAS were 0.6, 0.5, 1.3, 4.3, 0.2, and 0.1 ng/mL, respectively. This inverse relationship may stem from the fact that menstruation is an elimination route for PFAS. Additionally, while a lighter flow might seem desirable, it could make it more difficult to become pregnant [149].

Other endpoints included age at menarche (≤10, 11–13, 14+ years), menstrual cycle length (<25, 25–27, 28–31, >31 days), bleed length (<5, 5–7 or 8+ days), and a combined endpoint of “cycle length and flow.” For age at menarche, PFDA was associated with starting both earlier and later than the reference group of 11–13 years (~11% difference for both). No associations were found for any PFAS and menstrual cycle length or bleed length. Results for “cycle length and flow” combined endpoint did not align with findings from either measure individually.

Other health issues such as PCOS and diabetes are tied to both early and late menarche [150,151]. This is unsurprising considering the influence of EDCs on reproductive health and the non-monotonic responses commonly observed with EDCs. Early onset is associated with a number of health and/or psychosocial factors that may influence fertility indirectly, such as diabetes and riskier sexual behaviors [151,152]. Delayed onset technically reduces the fertility window, though with the average USA age of first-time childbearing being late 20s, this likely does not make an appreciable difference [153]. Another systematic review [154] on PFOA and PFOS found mostly no association with onset, though some studies showed delayed and one showed early onset. Other factors that may affect fertility are still being investigated for links to menarcheal timing, such as BMI [152,155] and endometrial cancer [156].

This cross-sectional study was unique in that it recruited only Black women from a predominantly Black city (Detroit, MI) (Study of Environment, Lifestyle and Fibroids (SELF)). Environmental racism is known to disproportionately affect minorities. Manufacturing or industrial sites are commonly placed in economically disadvantaged areas or communities of color [157,158]. PFAS and other EDCs have been found at higher levels in African American women than White women [140,159,160] and may contribute to reproductive issues. Higher infant mortality in non-Hispanic Black births [161,162] is likely attributed to structural racism [163,164]; at the same time, environmental exposures are an emerging area of research to help explain this phenomenon [161].

Heffernan et al. (2018) [81] looked solely at menstrual cycle irregularity in a prospective cohort study of case–controls. A total of 59 participants (30 PCOS cases; 29 controls) were recruited from an IVF clinic in the UK. The study focused on PCOS (described in the ovarian health section of this review); however, for this endpoint, they combined all participants together, irrespective of PCOS status. Serum samples were taken on Day 21 of the luteal phase before beginning IVF treatment. Irregular cycle was defined as variations in length for more than 7 days between cycles. They measured the differences of mean PFAS concentration in those with irregular or regular cycles. PFOS was significantly higher in the serum of patients with irregular menstrual cycle compared to those with regular cycle (irregular cycle geometric mean = 3.9 ng/mL; regular cycle geometric mean = 3.0 ng/mL; *p* = 0.011). None of the other PFASs studied (PFHxS, PFNA, PFOA) were associated. Geometric mean levels of these PFASs in all participants combined were 1.04, 0.57, and 2.39 ng/mL, respectively; means for the irregular and regular groups were not reported for these PFAS.

The final study on cycle characteristics (irregular, long, or short) came from 1977 women in a prospective population-based cohort (Norwegian Mother and Child cohort (MoBa)) [65]. Plasma samples were taken at 17–18 weeks of gestation. Cycle characteristics were self-reported via questionnaire. The analysis used percent change in plasma PFAS concentration for all outcomes. The authors were additionally interested in the effect parity and birth control use have on associations with PFAS. The authors acknowledge that the subgroup analyses of parity and contraceptive use were of small sample size and do not constitute strong evidence. The results are still reported here as this is an emerging field of research, and contraceptive use is understudied in general. Parity is a known factor that decreases PFAS levels as the placenta of each gestation readily transfers and bioaccumulates some of the mother’s PFAS and is then excreted [6,141,165]. Conversely, contraceptive use may lessen excretion leading to higher PFAS levels [166].

Women with irregular cycles showed a significant decrease in concentrations of PFDA (−17%), PFUnDA (−14%), PFNA (−9%), and PFOA (−7%). Median levels (ng/mL) for cases and controls were as follows: PFDA (0.19, 0.9), PFUnDA (0.32, 0.13), PFNA (0.63, 0.33), PFOA (3.3, 1.87). There was no effect of parity or contraceptive use. Overall, there was no significant association with long cycles, except in women who recently (last 12 months) used oral contraceptives, where long cycles where PFNA and PFUnDA were increased. There was no association with women who never used contraceptives or used them over a year ago. Overall, there was no significant association with PFAS and short cycles, except in parous women where PFHpS and PFOS were decreased.

As shown here, few recent, high-quality studies exist on these important reproductive endpoints. Three studies on irregular cycle [66,78,81] and two on cycle length [66,78] were overall inconclusive. However, both Wise et al. (2022) [78] and Zhou et al. (2017) [66] found PFAS modestly inversely associated with heavy bleeding in nearly every PFAS studied. Again, menstrual cycles are difficult to self-report without a rigorous study design that includes tracking materials for participants during their cycles and does not rely on memory. Larger sample sizes will help highlight small but significant effects.

The studies on PFOA and PFOS in both early menopause risk [62] and POI odds [75] were the most convincing due to their effect sizes (menopause early by 2 years, and up to 6× higher odds for POI) and reinforce the need for more work in these areas. As mentioned above, reverse causation must be considered in studies on menopause [134,141]. Both early menopause and POI are linked to serious health conditions including and beyond infertility [167,168]. Causes of each are multifaceted and include the environment [169]; thus, it is pertinent to continue researching PFAS in this area.

**Table 6:** Characteristics of menopause and menstruation studies.

Study	Study Type	Study Size (n)	Detected PFAS	PFAS Not Detected in 51% or More Samples	Outcomes	Sub-Outcomes	Media	Country
Ding et al. (2020) [62]	Prospective cohort	1120	**PFHxS**, **PFNA**, **n-PFOA**, **n-PFOS**, **Sm-PFOS**	PFDA, PFDoDA, Sb-PFOA, PFUnDA	Menopause	Incident natural menopause	Serum	USA
Heffernan et al. (2018) [81]	Case–control	Case: 30; Control: 29	**PFHxS**, **PFNA**, **PFOA**, **PFOS**	PFBS, PFDA, PFHpA, PFPeA, PFUnDA	Menstruation	Irregular cycle	Serum	UK
Singer et al. (2018) [65]	Prospective cohort	1977	PFDA, PFHpS, **PFHxS**, **PFNA**, **PFOA**, **PFOA**, PFUnDA	NA	Menstruation	Cycle regularity and length	Plasma	Norway
Wise et al. (2022) [78]	Cohort	1499	MeFOSAA, PFDA, **PFHxS**, **PFNA**, **PFOA**, **PFOS**, PFUnDA	NA	Menstruation	Age at menarche, cycle length and intensity	Plasma	USA
Zhang et al. (2018) [75]	Case–control	Cases: 120; Controls: 120	PFBS, PFDeA, PFDoA, PFHpA, **PFHxS**, **PFNA**, **PFOA**, **PFOS**, PFUA	PFOSA	Menstruation	Primary ovarian insufficiency	Plasma	China
Zhou et al. (2017) [66]	Cohort	950	PFBS, PFDeA, PFDoA, PFHpS, PFHxS, **PFNA**, **PFOA**, **PFOS**, **PFOSA**, PFUA	NA	Menstruation	Cycle regularity, length, and volume	Blood	China

Bold: common across all studies. Prefix n-: linear. Prefix Sm-: sum of branched isomers. Prefix Sb-: sum of branched. MeFOSAA: N-methylperfluorooctane sulfonamidoacetic acid. PFBS: perfluorobutane sulfonate. PFDA/PFDeA: perfluorodecanoic acid. PFDoA/PFDoDA: perfluorododecanoic acid. PFHpA: perfluoroheptanoic acid. PFHpS: perfluoroheptanesulfonic acid. PFHxS: perfluorohexane sulfonate. PFNA: perfluorononanoic acid. PFOA: perfluorooctanoic acid. PFOS: perfluorooctane sulfonate. PFOSA: perfluorooctane sulfonamide. PFPeA: perfluoro-n-pentanoic acid. PFUA/PFUnDA: perfluoroundecanoic acid.

### 3.6. Ovarian Health

Ovarian health is an important contributor to fertility. PCOS can limit ovulation [170,171], and endometriosis frequently co-occurs with infertility [172]. The origins of either condition are incompletely understood. Existing works have focused on the relationship between PFAS and hormones to link endocrine disruption or hormone imbalance to fertility or ovarian issues [97,173,174]. Here, we decided to cover PFAS with regards to the less-studied female fertility endpoints of clinically diagnosed PCOS and endometriosis.

PCOS is defined by having at least two of the following signs: irregular periods, hyperandrogenism, and/or cysts on the ovaries that can hinder egg release [175].

Many factors influence PCOS status and severity: insulin resistance, androgen levels, and adiposity are a few [176,177,178]. Higher adiposity is seen in as much as 80% of women with PCOS in the US. Rates are lower in other countries, suggesting environmental factors play a role in the USA [179]. Endometriosis is a condition in which endometrial-type tissue grows outside of the uterus. This tissue does not shed during menstruation and can build up causing cysts or scarring, the consequence of which can be reduced fertility.

A previous review included the papers covered here, and additionally one earlier study on PCOS and endometriosis each. Rickard et al. (2022) [29] noted that Vagi et al. (2014) [180] found positive associations between PFAS exposure and PCOS, and that Campbell et al. (2016) [181] used NHANES data to show mean levels of PFNA, PFOA, and PFOS were higher in women with endometriosis. Another study found an insignificant link to increased odds between PFAS and endometriosis [182].

In this systematic review, three identified studies met our inclusion criteria (Table 7). Two studies focused on PCOS (one in the UK, one in China) and one on endometriosis in China. All three were case–control studies recruiting from IVF centers, and PFOA and PFOS were found to be the highest concentrations of any PFAS. PFOA and PFOS were found at median levels of 2.39 and 3.46 ng/mL (respectively) in the UK PCOS study, and 5.07 and 4.05 ng/mL (respectively) in the Chinese PCOS study. The Chinese endometriosis study did not report the overall medians; median in cases was 14.67 and 6.4 ng/mL (respectively).

#### 3.6.1. PCOS

Heffernan et al. (2018) [81] was an age- and weight-matched case–control study (30 PCOS cases, 29 controls) that recruited from the Hull IVF Unit in the UK and sampled serum and follicular fluid for 13 PFAS. Serum samples were taken on Day 21 of the luteal phase before beginning IVF treatment. PCOS was defined using the Rotterdam criteria—PCOS was present if any two out of three criteria were met: menstrual disturbance (oligo- and/or anovulation), clinical and/or biochemical signs of androgenism or polycystic ovaries on ultrasound [175]. The authors simply compared the mean difference of PFAS concentration between cases and controls, and found PFOS was significantly higher in the serum of PCOS patients: 3.9 ng/mL in cases and 3.1 ng/mL in controls. No associations were found for the three other detected PFASs (PFHxS, PFNA, PFOA) in serum. There was no significant difference in cases in follicular fluid concentration of any PFAS. PFAS concentrations in serum and follicular fluid were highly correlated (R^2^ > 0.95), suggesting the more easily obtainable serum collection is sufficient for studying this outcome. However, as blood–follicle transfer efficiencies may differ based on the PFAS characteristics [183], using both media was a strength of this study. Another strength was its power calculation and recruitment of more participants than the minimum indicated.

Wang et al. (2019) [184] was a case–control study (180 cases, 187 controls) that recruited and took plasma samples from the Center for Reproductive Medicine of Shandong University in China. They found no significant mean differences in PFAS concentration in plasma between controls and PCOS-related infertility cases. Odds ratios for PCOS insignificantly decreased with increasing PFUA concentrations in Tertiles 2 and 3 (aOR = 0.69, 0.29; *p* = 0.38, 0.06), but the overall inverse trend was significant (*p* = 0.03). Median PFUA levels in cases was 0.41 ng/mL and 0.39 ng/mL in controls. ORs significantly increased in a dose-dependent manner with increasing PFDoA concentration in Tertiles 2 and 3 (aOR = 2.36 (1.12, 4.99); 3.04 (1.19, 7.67) and the direct trend was significant across tertiles (*p* = 0.01). Median PFDoA level in cases was 0.24 ng/mL and 0.23 ng/mL in controls.

Both Heffernan et al. (2018) [81] and Wang et al. (2019) [184] used the same criteria for diagnosing PCOS (Rotterdam ESHRE/ASRM revised 2003 consensus [175]) and infertility (inability to become pregnant after having unprotected intercourse for more than one year), used the same methods for PFAS measurement (HPLC-MS/MS), and found comparable serum PFAS levels. Heffernan et al. (2018) [81] collected serum at a very specific time in all participants (Day 21 of luteal phase), while exact collection timing in Wang et al. (2019) [184] was unspecified. Heffernan et al. (2018) [81] did not measure PFDoA and did not detect PFUA, and thus these chemicals could not be compared across the two studies on PCOS.

#### 3.6.2. Endometriosis

Wang et al. (2017) [72] was a case–control study (157 cases, 178 controls) that recruited and took plasma samples from the Women’s Hospital Affiliated to Zhejiang University School of Medicine in China. Plasma was collected at enrollment. Endometriosis was confirmed by surgical visualization using laparoscopy. Infertility was defined as failure to conceive after >12 months of attempts. Across multiple adjusted models, highest tertile PFBS was associated with increased odds of endometriosis-related infertility (aOR range: 3.04–3.41), and PFNA was associated with decreased odds (range: 0.45–0.52). PFBS levels were 0.091 ng/mL in cases and 0.089 ng/mL in controls. PFNA levels were 1.05 ng/mL in cases and 1.20 ng/mL in controls. PFHpA levels lowered odds in a partially adjusted model (aOR = 0.48); when analyses were restricted to those without a history of pregnancy or without other gynecologic pathologies, the association disappeared. PFHpA levels were 0.09 ng/mL in cases and 0.10 ng/mL in controls. This brings up the point that PFAS may exacerbate existing gynecologic conditions rather than being involved in their origin.

**Figure 6:**
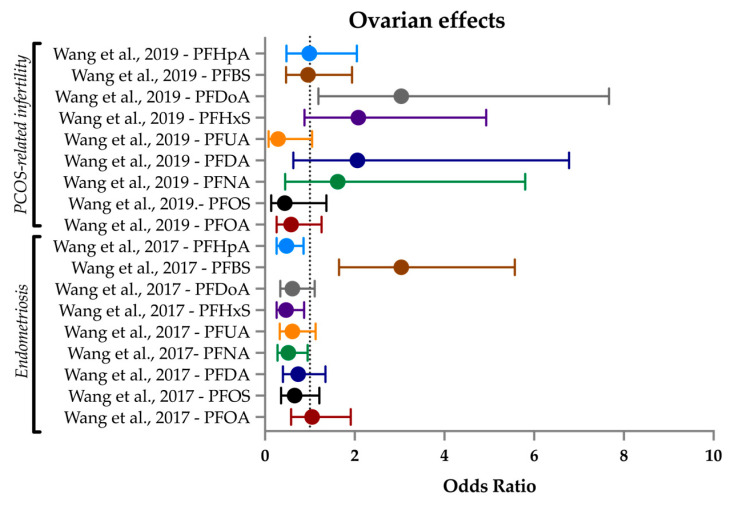
Forest plot of odds and risk ratios of ovarian health effects (top: endometriosis; bottom: PCOS-related infertility) with increasing PFAS levels from two studies. PFHpA: perfluoroheptanoic acid. PFBS: perfluorobutanesulfonic acid. PFDoA: perfluorododecanoic acid. PFHxS: perfluorohexane sulfonic acid. PFUA: perfluoroundecanoic acid. PFDA: perfluorodecanoic acid. PFNA: perfluorononanoic acid. PFOS: perfluorooctanesulfonic acid. PFOA: perfluorooctanoic acid. Wang et al., 2019 [184]. Wang et al., 2017 [72].

Altogether, there is limited yet suggestive evidence for PFAS’ influence on the odds of PCOS and endometriosis in women who seek IVF (Figure 6). PFDoDA and PFBS—or more likely lifestyle factors that affect exposure of these less common PFAS—moderately increased the odds of PCOS or endometriosis contributing to infertility. PFOS was significantly associated with PCOS in one study, and not in the other. Reverse causation should be considered in both conditions. Menstrual bleeding is generally heavier with endometriosis, and PCOS is characterized by irregular periods which can lead to less overall elimination. Additionally, higher BMI is commonly present in PCOS; higher adiposity may disrupt pharmacokinetics of elimination of PFAS [185]. More research is clearly required to take a more confident stance on PFAS and PCOS- or endometriosis-related infertility. Future high-quality work should follow common guidelines for diagnosis such as the Rotterdam criteria [175] along with pathologic evidence when possible and should include lesser-studied PFAS such as PFBS, PFUA, and PFHpA.

**Table 7:** Characteristics of ovarian health studies.

Study	Study Type	Study Size (n)	Detected PFAS	PFAS Not Detected in 51% or More Samples	Outcomes	Sub-Outcomes	Media	Country
Heffernan et al. (2018) [81]	Case–control	Cases: 30; Controls: 29	**PFHxS**, **PFNA**, **PFOA**, **PFOS**	PFBS, PFDA, PFHpA, PFPeA, PFUnDA	Ovarian health	PCOS	Serum, follicular fluid	UK
Wang et al. (2017) [72]	Case–control	Cases: 157; Controls: 178	PFBS, PFDA, PFDoA, PFHpA, **PFHxS**, **PFNA**, **PFOA**, **PFOS**, PFUA	PFOSA	Ovarian health	Endometriosis-related infertility	Plasma	China
Wang et al. (2019) [184]	Case–control	Cases: 180; Controls: 187	PFBS, PFDA, PFDoA, PFHpA, **PFHxS**, **PFNA**, **PFOA**, **PFOS**, PFUA	PFOSA	Ovarian health	PCOS-related infertility	Plasma	China

Bold: common across all studies. PFBS: perfluorobutane sulfonate. PFDA: perfluorodecanoic acid. PFDoA: perfluorododecanoic acid. PFHpA: perfluoroheptanoic acid. PFHxS: perfluorohexane sulfonate. PFNA: perfluorononanoic acid. PFOA: perfluorooctanoic acid. PFOS: perfluorooctane sulfonate. PFOSA: perfluorooctane sulfonamide. PFPeA: perfluoro-n-pentanoic acid. PFUA/PFUnDA: perfluoroundecanoic acid.

### 3.7. Sperm Health

We included the male reproductive endpoint of sperm quality as male health outcomes are often overlooked in the PFAS literature, save testicular cancer [35,186]. Males are also understudied in the infertility space, despite the fact that about half of infertility cases can be attributed to males, with up to 30% of male-factor infertility being idiopathic [187]. One explanation for idiopathic male infertility is exposure to environmental contaminants [188,189]; thus, it is important to examine sperm health to achieve a well-rounded review of PFAS’ effects on reproduction. Male infertility is an indicator of overall health; further-reaching is the fact that co-morbidities with infertility are often transgenerational in nature [35]. Preconceptual environmental exposure to contaminants can alter the sperm epigenome which controls developmental programming of the resulting embryo [190]. This germline program can be transmitted to subsequent generations even in the absence of exposure [38]. With so much at stake, it is a public health imperative to review sperm as a sentinel marker of current and future well-being.

The literature search identified three cross-sectional studies: two from China and one from the Faroe Islands (Table 8). All samples were collected at fertility clinics. The five shared outcomes between the two studies are semen volume, sperm motility, concentration, count, and morphology. Pan et al. (2019) [69] additionally examined DNA instability. All measured PFAS in blood, and two included semen. Combined, 13 PFAS were investigated; PFOA and PFOS were shared among all studies. Median PFOA levels ranged from 0.23 to 0.74 ng/mL in semen and 2.8 to 8.57 ng/mL in blood/serum. Median PFOS levels ranged from 0.1 to 3.9 ng/mL in semen and 8.38 to 96 ng/mL in blood/serum. Every PFAS detected in blood/serum was also detected in semen. The highest median level of PFAS found in serum, blood, or semen was PFOS (19.5, 96, 3.9 ng/mL, respectively).

Song et al. (2018) [67] collected blood and semen of 103 men recruited from an infertility clinic at the Third Affiliated Hospital of Sun Yat-sen University in China in their cross-sectional study. This clinic is in the most populous province of China, and specifically in the Pearl River Delta, a region of heavy industry and contamination [191] where the general population is more highly exposed to PFAS than, for example, the third most populous province of China [192]. Blood and semen samples were taken on the same day. They excluded those with reproductive diseases. Progressive motility of sperm was analyzed using WHO guidelines of class A + B [193]. Sperm concentration was measured via hemocytometry.

Correlations between PFAS in semen (and blood) and progressive motility were reported. In semen, all correlations were negative, and almost all significantly so. For the sum of total PFAS and motility, r = −0.495 (*p* < 0.001). Individual PFAS had weak correlations ranging from r = −0.35 (PFHxA; median 3.4 ng/mL; *p* < 0.01) to r = −0.20 (PFOS; median 3.9 ng/mL; *p* < 0.05). In blood, correlations with motility were not in directional agreement and were sporadically significant. For sperm concentration in semen, there were no significant findings with any PFAS.

Pan et al. (2019) [69] recruited 664 men from the infertility clinic at Nanjing Jinling Hospital in China for their cross-sectional study. Semen and serum samples were taken on the same day. The authors attempted to represent the general population with their sample, and thus recruited a heterogeneous group of men visiting the clinic for either male- or female-factor infertility. They did not distinguish between the two groups (fertile or infertile men) in their analysis. They excluded those with severe reproductive diseases and one fluorochemical plant worker. Many parameters of semen or sperm health were measured: motility, volume, count, concentration, morphology, and DNA instability. Sperm concentration and progressive motility were measured with computer-aided sperm analysis system (CASA). Sperm count was calculated by concentration times volume. Morphology was judged from stained slides using WHO criteria (edition not explicitly specified, but is likely 5th edition [194]. DNA instability was probed by a chromatin stability assay measuring both DNA fragmentation (percentage of sperm with damaged DNA (DNA fragmentation index (DFI%)) and immature chromatin (percentage of sperm with high DNA stainability (HDS%)) via flow cytometry. *p*-values for all models were FDR-adjusted.

Count, concentration, and volume were largely unaffected. 6:2 Cl-PFESA and PFDA showed a significant increase in volume at the highest quartile for both serum and semen, but the trend was not significant. Logically, both chemicals were then associated with a decrease in sperm concentration, again not significantly across quartiles. No significant findings were reported for morphology. For motility, linear regressions for nearly all PFAS revealed significant (*p* < 0.05) strong negative associations ranging from B = −2.4 (−3.9, −0.8) (PFOA) to −1.6 (−2.8, −0.4) (6:2 Cl-PFESA); 6:2 Cl-PFESA remained significant in quartile regressions for Q4 for both serum and semen (B = −3.94 (−7.04, −0.83)); p for trend not significant). Median 6:2 Cl-PFESA concentration in serum was 3.36 ng/mL and 0.04 ng/mL in semen.

DNA stability was a sensitive endpoint. All PFASs (except PFOS and PFOA) were significantly positively associated with increased immature chromatin (HDS%) in both linear and quartile regressions, in both serum and semen. Quartile trends were often significant overall as well. Notably, 6:2 Cl-PFESA showed a significant monotonic dose response across all quartiles (serum p-trend = 0.01; semen p-trend non-significant). All PFAS (PFUnDA not included) in semen were associated with an increase in damaged DNA (DFI%) in linear regression, with many remaining significant (*p* < 0.05) for the trend in quartile regression (PFUnDA included). B-values ranged from 0.08 (95% CI: 0.026, 0.135; 6:2 Cl-PFESA) to 0.136 (95% CI: 0.064, 0.209; PFOA) in linear regression. Median PFOA level in semen was 0.23 ng/mL. In quartile regression, B-values ranged from 0.16 (95% CI: 0.01, 0.30; PFUnDA) to 0.25 (95% CI: 0.10, 0.40; PFOS). Median PFUnDA and PFOS levels in semen were 0.016 and 0.097 ng/mL, respectively. PFASs seem to have an effect on sperm DNA only when PFAS is measured in semen. In serum PFAS measurements, there were no significant findings for either measure of DNA stability in sperm.

An important strength of this paper was its FDR adjustment for *p*-values in all models, which increases the weight of the evidence. PFAS did not strongly affect sperm parameters in this study, save for DNA stability. This endpoint should be included in future sperm health population studies. Although there was a moderate to very strong correlation between PFAS detected in semen and serum (r-values range from 0.58 (PFHxS, *p* < 0.001) to 0.83 (6:2 Cl-PFESA, *p* < 0.001)), associations were uncovered more often with PFAS levels in semen rather than serum. Both are easily obtainable non-invasive media and should be included in future studies.

Petersen et al. (2018) [61] used a population-based cross-sectional study of 263 men aged 24–26 years recruited from the entire population of men born between 2007 and 2009 in the Faroe Islands. PFAS was quantified in serum only. The Faroe Islands contain a genetically homogeneous population and have high PFAS exposure through their diet which includes seafood; this seafood may include traditional pilot whale meat containing bioaccumulated PFAS [195,196,197]. Semen and serum samples were taken on the same day. As in Pan et al. (2019) [69], the authors measured motility, volume, count, concentration and morphology. Concentration and count were measured using a hemacytometer. Motility was classified according to WHO guidelines (progressive motility = class A + B) [198], and morphology was judged according to “strict criteria” used at the University Department of Growth and Reproduction, Rigshospitalet, University of Copenhagen. The criteria were not well-cited but likely refer to the 1990 standards set forth by Menkveld et al. (1990) [199]. PFOA and PFOS were detected in serum (median levels 2.8 and 19.5 ng/mL, respectively) and the results centered on these two PFAS. PFNA, PFHxS, and PFDA were also detected above the LOQ but were not included in the analyses. There were no significant findings for any PFAS with any measure of sperm health.

**Figure 7:**
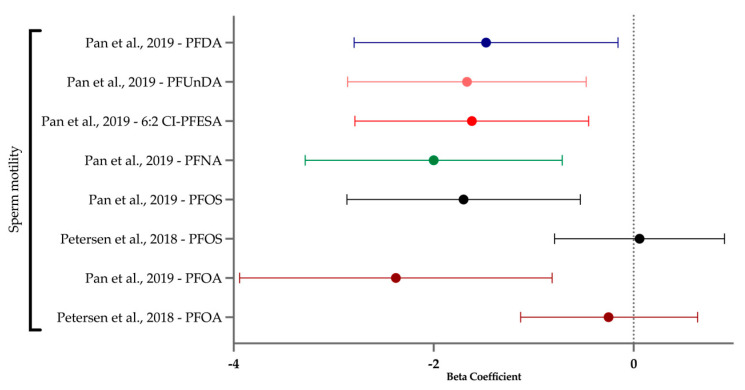
Forest plot of beta coefficients for sperm health effects with increasing PFAS levels in semen from two studies. Top panel: DNA stability (decreasing); DNA fragmentation index. Bottom panel: sperm motility (% motile sperm). PFDA: perfluorodecanoic acid. PFUnDA: perfluoroundecanoic acid. 6:2 Cl-PFESA: 6:2 chlorinated polyfluorinated ether sulfonate. PFNA: perfluorononanoic acid. PFOS: perfluorooctanesulfonic acid. PFOA: perfluorooctanoic acid. Pan et al., 2019 [69]. Petersen et al., 2018 [61].

In summary, studies examining PFAS and sperm health found negative associations with sperm motility and DNA stability, and no associations for volume, concentration, count, or morphology when PFAS were measured in semen (not serum) (Figure 7). All PFAS were associated with DNA damage (6:2 Cl-PFESA, PFDA, PFNA, PFOA, PFOS, and PFUnDA) [69]. The importance of the sperm genome and epigenome in reproductive health cannot be overemphasized. It is reasonable to recommend more studies, given the moderate effects shown here. Interestingly, PFOA and PFOS were the only two PFAS to not affect chromatin maturity [69], underscoring the need for more research into non-legacy PFAS/PFAS alternatives. Future studies should strongly consider including phenotypic endpoints as done in Pan et al. (2019) [69]. Semen is an easily accessible PFAS depot with higher sensitivity to sperm parameters; however, equipment capable of reliably reading into the fg/mL range is necessary for future PFAS analysis in semen.

### 3.8. Fetal Growth

Fetal growth is included in this review as a holistic part of maternal health. In utero growth is determined by both maternal and fetal factors. As the developmental origins of health and disease hypothesis posits, there are critical windows during development that can affect fetal outcomes both acutely and later in life [200]. Gestation is a particularly sensitive period for exposures. Environmental contaminant exposure during this period can disrupt the ongoing cellular programming during fetal development resulting in adverse structural or functional changes. PFAS readily cross the placental barrier, are detected in placenta [95], and indeed accumulate in fetal organs [3]. Newborn (ex utero) morphometrics such as head circumference and birth weight have been well studied; however, in utero growth has not received the same attention. For example, a recent meta-analysis of 46 studies on newborns showed PFOS and PFDoDA inversely associated with head circumference and multiple PFAS linked to low birth weight [107]. PFOA and PFOS have been modestly linked to low birth weight in earlier meta-analyses as well [201,202]. Head circumference is a proxy for brain size, and small head circumference at birth is related to intellectual deficiencies in childhood and anxiety in adulthood [203]. Low birth weight is associated with adverse effects later in life such as asthma and insulin resistance [204,205]. Understanding whether or how morphometrics are affected in utero helps define critical windows during gestation and is a necessary supplement to the body of evidence on PFAS and newborn measurements.

Only two identified cohort studies approach in utero growth (Table 9). Both Costa et al. (2019) (Spain) [73] and Ouidir et al. (2020) (USA) [77] employ multiple sonography appointments spanning gestational Weeks 12–40. Both shared measures of head size (biparietal diameter (BD)), appendicular skeleton length (femur length (FL)), and overall body size (estimated fetal weight (EFW), abdominal circumference (AC)). Ouidir et al. (2020) [77] detected eight PFASs (PFOS, PFOA, PFHxS, PFNA, PFUnDA, PFDA, N-MeFOSAA, PFDoDA). Costa et al. (2019) [73] examined four of these PFAS as well (PFOS, PFOA, PFHxS, PFNA). The PFAS with the highest concentration in plasma was PFOS in both studies (5.16 and 6.05 ng/mL, respectively). PFOA levels were 2 and 2.35 ng/mL, respectively.

Costa et al. (2019) [73] enrolled 1230 women in the Infancia y Medio Ambiente (Environment and Childhood) (INMA) Project cohort. Plasma samples were taken at the end of the first trimester (average 13.5 weeks of gestation). Ultrasounds were taken at the first, second, and third trimesters (Weeks 12, 20 and 34) by specialized obstetricians for four measures of fetal growth: BD, AC, FL, and EFW. EFW was calculated using the Hadlock formula [206]. Median levels of PFHxS, PFNA, PFOA, and PFOS were 0.58, 0.65, 2.35, and 6.05 ng/mL, respectively. Median PFAS levels were reported for the aggregate and not delineated by smokers vs. non-smokers.

There were no significant findings in the main analysis. Only when restricting to smokers did inverse associations with PFNA and PFOA appear at 20 weeks. Authors found that for every two-fold increase in PFNA and PFOA concentration, there was a change in FL of −6.3% (95% CI: −11.9, −0.5) and –6.8% (95% CI: −12.4, −1.0), respectively. By 34 weeks, there were no significant differences for these PFASs. Similarly, EFW for smokers was lower in Week 20 with increasing PFNA concentrations (−6% change (95% CI: −11.6, −0.3)); however, by Week 34, there were no associations. In smokers only, positive associations with BD were revealed at 34 weeks with PFHxS and PFOS (+6.8% (95% CI: 0.5, 12.9) and +6.3% (95% CI: 0.1, 12.3), respectively). There were no associations for AC. Overall, there were a few significant findings. Some differences in smokers were revealed in week 20, but by week 34 these dissipated, except for a persistent ~6.5% increase in BD with PFHxS and PFOS.

The other study in this review did find associations between PFAS and fetal growth in their main analysis. Ouidir et al. (2020) [77] enrolled 2284 women from 12 study sites across the USA for the National Institute of Child Health and Human Development Fetal Growth Studies–Singleton cohort. Plasma samples were taken at enrollment which ranged from gestational Week 8 to 14. The participants had five ultrasound appointments, with the first between Weeks 16 and 22. Time between appointments was 4–8 weeks [207]. Fourteen fetal measurements were taken at each session by trained sonographers. They focused their reporting on head size (head circumference (HC)), AC, and FL. We additionally report findings for BD and EFW in order to better compare findings with BD and EFW in Costa et al. (2019) [73]. In the overall sample, median levels of PFDA, PFHxS, PFNA, PFOA, and PFOS were 0.25, 0.71, 0.77, 2, and 5.16 ng/mL, respectively. A strength of this study was its use of FDR and a very low ROB score (9).

Authors found no strong associations with AC or HC. Some associations with PFHxS, PFNA, PFOA, and PFOS were weak to moderate for these outcomes, but did not hold when controlling for false discovery rate. For example, PFDA, PFNA, and PFOS were moderately associated with an increase in AC (B = 0.43, 0.28, 0.38, respectively; *p* < 0.05). PFDA was weakly associated with an increase in head circumference (B = 0.21 *p* < 0.05), while PFHxS and PFOS were weakly associated with a decrease in head circumference (B = −0.22 (*p* < 0.05), −0.27 (*p* < 0.01), respectively).

Another measure of head size, however, did pass the FDR threshold: PFNA was weakly associated with a significant decrease in BD (B = −0.12, FDR < 0.001). Other BD findings did not pass FDR and contradict the head circumference findings: PFHxS was strongly associated with an increase in BD (B = 0.7, *p* < 0.05) and PFDA with a slight decrease (B = −0.08, *p* < 0.01).

PFHxS and PFOA had reliably positive yet weak associations with femur length (B= 0.12 (FDR < 0.001); 0.13 (FDR < 0.001), respectively).

EFW showed a strong association with PFDA. PFDA was associated with an increase in EFW (B = 6.11, *p* < 0.01); however, this did not pass FDR.

**Figure 8:**
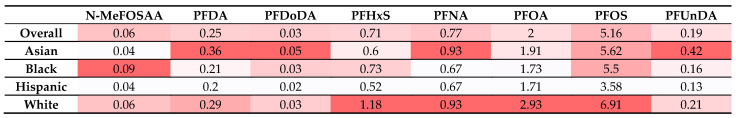
For each PFAS, median level (ng/mL) across ethnicities (concentrations are compared by column, not row). Color darkens with increasing median concentration. Me-FOSAA: N-methylperfluorooctane sulfonamidoacetic acid. PFDA: perfluorodecanoic acid. PFDoDA: perfluorododecanoic acid. PFHxS: perfluorohexane sulfonate. PFNA: perfluorononanoic acid. PFOA: perfluorooctanoic acid. PFOS: perfluorooctane sulfonate. PFUnDA: perfluoroundecanoic acid.

This study was notable for delineating every endpoint by race/ethnicity and by female/male fetuses, an important step in addressing potential disparities in exposure and reproductive outcomes. Median PFAS levels are reported separately for each group as well (see Figure 8).

**Figure 9:**
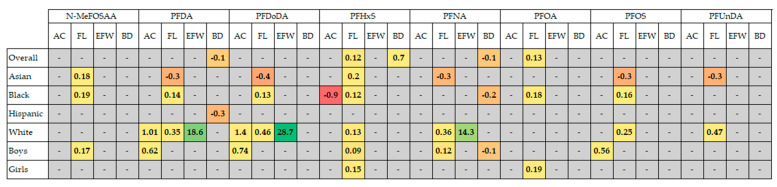
Heatmap of beta-values at FDR < 0.05 for measures of fetal growth, broken down by group. Warmer colors (orange, red) indicate lower values, yellow indicates mid-range values, and green indicates higher values. AC: abdominal circumference, FL: femur length, EFW: estimated fetal weight, BD: biparietal diameter. Head circumference not included (not significant for any ethnicity). Me-FOSAA: N-methylperfluorooctane sulfonamidoacetic acid. PFDA: perfluorodecanoic acid. PFDoDA: perfluorododecanoic acid. PFHxS: perfluorohexane sulfonate. PFNA: perfluorononanoic acid. PFOA: perfluorooctanoic acid. PFOS: perfluorooctane sulfonate. PFUnDA: perfluoroundecanoic acid.

Ethnicity was self-reported as Hispanic (27.8%), White (26.5%), Black (25.8%), or Asian (19.9%). For Hispanics, there was a significant negative association with BD and PFDA (B = −0.25, FDR < 0.05). For White people, three PFAS had strong significant positive associations with EFW (PFDA (B = 18.58, FDR < 0.01), PFNA (B = 14.3, FDR < 0.1), PFDoDA (B = 28.7, FDR < 0.001)); it was similar for AC (PFDA (B = 1.01, FDR < 0.05), PFDoDA (B = 1.40, FDR < 0.05)). In contrast, for Black people, there was an inverse association with PFHxS and AC (B= −0.88, FDR < 0.05). Black people also showed a negative association between PFNA and BD (B= −0.20, FDR < 0.05). Both White and Black people showed significant positive associations with FL with an FDR of <0.10 or better. White people had beta values ranging from 0.25 or less (PFHxS, PFOS; in increasing order) or 0.35 to 0.50 (PFDA, PFNA, PFDoDA, PFUnDA; in increasing order). Black people’s beta values ranged from 0.12 to 0.19 (PFHxS, PFDoDA, PFDA, PFOS, PFOA, N-MeFOSAA; in increasing order). Interestingly, in Asian people, this was generally reversed (beta values from –0.25 to –0.50: PFNA, PFDA, PFUnDA), PFOS, PFDoDA; decreasing order), though N-MeFOSAA and PFHxS had positive beta values (0.18 and 0.20, respectively). See Figure 9 for a summary of these values.

A total of 55% of fetuses were male and 45% female. Males were more sensitive to PFAS than females, though PFASs seem to have had a protective effect on growth. AC and BD were significantly affected in males but not females (AC: PFOS (B = 0.56), PFDA (B = 0.62), PFDoDA (B = 0.74); BD: PFNA (B = −0.13)). Femur length was affected in both for PFHxS (male: B = 0.09; female: B = 0.15); additionally, N-MeFOSAA for males (B = 0.17) and PFNA (B = 0.12), and PFOA (B = 0.19) in females. EFW was not affected in either sex.

Beyond what can be directly compared between the two studies, Ouidir et al. (2020) [77] examined other measures of cranium size (cerebral width, inner and outer orbit diameter, occipital–frontal diameter) and appendicular skeleton lengths (tibia, fibula, radial, ulnar, humerus, foot). For cranium size in the overall cohort, cerebral width and occipital–frontal diameter were null. Four PFAS were positively associated with inner orbit diameter with an FDR of <0.05 (PFHxS and PFNA (B = 0.08), PFDA (B = 0.11), PFOA (B = 0.13)), and two of those were negative for outer orbit diameter (PFHxS and N-MeFOSAA, B= −0.13). Hispanic participants were the least affected in the cranium measurements, and White participants were affected the most. There was no clear difference between male and female fetuses. For appendicular skeleton length in the overall cohort, every measure (six of six) showed only positive associations with at least one and as many as four PFAS (of the eight measured). Hispanic and Asian participants defied the overall trend, showing either mainly negative or no associations for all measures. There was no clear difference between male and female fetuses.

Findings in this study are mostly positive (albeit not strong), indicating higher PFAS exposure may increase fetus size or protect against small fetus size. Other studies have found similar links between PFAS and “better health”, and have noted this correlation may be partially explained by socioeconomic status (SES) [208]. Other studies of PFAS and determinants of health have shown higher earners may have more access to PFAS-containing goods or diet [209], and better fetal health is often associated with higher SES [210]. Ouidir et al. (2020) [77] did control for SES by proxy of maternal education level. The idea that higher earners have greater access to PFAS-containing goods in interesting because, although PFAS are found in higher-cost consumer items such as Gore-Tex^TM^ clothing, they are also found everywhere, including in food wrappings that people with lower SES may contact in lieu of, for example, fresh produce without wrappings. Generally, lower SES comes with the lack of “freedom from” environmental chemicals that higher earners can more easily avoid; see BPA-free baby products, organic produce, housing away from industrial areas, etc. The occasional associations seen here between PFAS and fetal growth may correlate, and the correlation is yet to be determined. In fact, Ouidir et al. (2020) [77] noted their findings in utero are inconsistent with anthropometric measures of newborns from the same NICHD cohort [211].

Overall, associations between plasma PFAS concentrations and fetal growth outcomes reported in both large studies are inconsistent. Some PFAS compounds were linked to minor changes in femur length, biparietal diameter, and estimated fetal weight, but these findings were inconsistent. Smoking, race/ethnicity, and fetal sex may be relevant mediators for this endpoint and should be included in future studies. A study since this systematic search found PFOA stunted in utero growth, driven by higher stress levels in mothers [212], emphasizing the interplay between environmental chemicals and the maternal environment. It is thus recommended to include stress, smoking, and the multitude of factors in the exposome.

## 4. Conclusions

The impetus for this systematic review was to provide a rigorous, reproducible, and informative summary of the risks of background level PFAS exposure to reproductive outcomes in the general population. Exposures during the critical period of pregnancy affect both mother and fetus, and can have long-lasting effects. This review is one response to the public health imperative to study xenobiotics and reproduction. It can be used to inform those dealing with allocating valuable, scarce, and competing resources: grassroots organizations, government, industry, academia, and society of the realistic risks posed by ambient PFAS.

In summary, there was no effect on all-cause fertility. There was weak to moderate evidence for increased preterm birth, miscarriage, PCOS- and endometriosis-related infertility, decreased sperm motility, and DNA health. There was limited yet suggestive evidence for PFAS and menopause, primary insufficiency, and inconsistent results for fetal growth. Table 10 summarizes the conclusions of all endpoints.

This review process would have been strengthened by including more recent studies. The literature search parameters were determined by available resources. The data aggregation presented in the forest plots would have benefitted from more studies reporting data in the same format (e.g., beta values and odds ratios with 95% CIs) so as to allow comparison. The included studies were high quality and many had not been systematically reviewed previously.

Environment played a salient role in many studies, most clearly evidenced by the obvious differences and sometimes reversals in findings between the large study sites of Beijing and Yantai conducted in the same method by the same group. Further, only in smokers did Costa et al. (2019) [73] reveal reductions in fetal growth mid-term, though these changes did not persist in late gestation.

One proxy for environment included in a handful of studies was race/ethnicity. This is a worthwhile step in addressing disparities in exposure and reproductive outcomes. However, like much of the scientific literature, the minority categories do not follow current National Academies of Sciences, Engineering, and Medicine (NASEM) guidelines, which warn against, among other things, grouping nationalities into ethnicities as in [197] (Chinese and Japanese become “Asian”), [213]. Though NASEM is intended for population genetics studies, their best practices can improve all science. A more appropriate grouping could have been by determinants known to be associated with PFAS, rather than race/ethnicity. Future studies should continue to include ethnicity or ancestry only if explicitly defining the criteria for those groupings.

It will behoove future studies to use power analyses to plan cohorts large enough to uncover subtle effects of background exposure, measure and report on lesser-studied PFAS, include fetal sex as a modifier, supplant the use of self-recall in surveys with more reliable tools such as menstrual tracking apps in real time, take care to account for reverse causation, record other exposome factors such as smoking, stress, or other contaminants, and supply open access raw datasets and journals. Open access datasets will facilitate comparison across studies using, for example, forest plots of the same test statistic. Open access publication is essential for reaching every stakeholder, including community members.

Considering all evidence presented above, the authors reason that background levels of PFAS pose a slight yet significant threat to reproductive health in men and women, and steps should be taken to reduce exposure when possible.

## Figures and Tables

**Table 1:** Search strategy for systematic review.

Database	Search Terms
PubMed	(“PFAS” [Title/Abstract] OR “perfluorinated” [Title/Abstract] OR “polyfluoroalkyl” [Title/Abstract] OR “perfluoroalkyl” [Title/Abstract] AND (reproduct * OR fertile * OR fecund * OR infertile * OR subfertil * OR pregnan * OR menstr * OR menopaus * OR menarche OR endometriosis OR PCOS OR polycystic ovarian syndrome)
Embase	PFAS AND (reproduct * OR fertile * OR fecund * OR infertile * OR subfertil * OR pregnan * OR menstr * OR menopaus * OR menarche OR endometriosis OR pcos OR (polycystic AND ovarian AND syndrome)) and (2017:py OR 2018:py OR 2019:py OR 2020:py OR 2021:py OR 2022:py)
Web of Science	(AB = (PFAS OR polyfluoroalkyl OR perfluoroalkyl OR perfluorinated) AND ALL = (reproduct * OR fertile * OR fecund * OR infertile * OR subfertil * OR pregnan * OR menstr * OR menopaus * OR menarche OR endometriosis OR PCOS OR polycystic ovarian syndrome)) Refined by: Language: English, Timespan: 2017–2022, Document type: Article
HERO	Search For: “PFAS” (match all words) Search For: “reproduct *, fertile *, fecund *, infertile *, subfertil *, pregnan *, menstr *, menopaus *, menarche, endometriosis, PCOS, polycystic ovarian syndrome (match any word) Years: “2017 to 2022”

* is the Boolean wildcard returning all words that start with the stem preceding the asterisk.

**Table 2:** PECO statement.

**Population**	Humans (mothers, men, and women in IVF clinics, young women, fetuses)
**Exposure**	Any PFAS chemical
**Comparator**	Lowest exposed in group compared to more highly exposed in group (i.e., lowest tertile vs. highest tertile), continuous levels, or comparing PFAS levels in groups with or without outcome of interest.
**Outcome**	Reproductive endpoints (fertility, preterm birth, miscarriage, menstruation and menopause, ovarian health, sperm health, and in utero fetal growth)

**Table 3:** Characteristics of fertility studies.

Study	Study Type	Study Size (n)	Detected PFAS	PFAS Not Detected in 51% or More Samples	Outcomes	Media	Country
Bjorvang et al. (2021) [82]	Cohort	50	**PFDA**, PFDoA, PFHpA, PFHxA, **PFHxS**, **PFNA**, **PFOA**, **PFOS**, **PFUnA**	NA	Ovarian reserve; Growing, Healthy or Atretic Follicle Density; infertility (TTP > 12 months)	Serum	Sweden
Bjorvang et al. (2022) [80]	Cohort	185	**PFDA**, PFHpA, PFHxA, **PFHxS**, **PFNA**, **PFOA**, **PFOS**, **PFUnA**	**PFDoA**	Basal antral follicle count, Ovarian sensitivity index, Average embryo score, at least one top quality embryo, Clinical pregnancy from fresh or fresh/frozen transfer, Live birth from fresh or fresh/frozen transfer	Plasma, follicular fluid	Sweden
Wang et al. (2021) [71]	Prospective cohort	305	PFBA, PFBS, **PFDA**, PFHxA, **PFNA**, **PFOA**, **PFOS**, **PFHxS**	PFDoA *, PFHpA, PFPeA *, **PFUdA** *	Clinical pregnancy failure, hCG test negative 14 d after transfer	Serum	China
Wise et al. (2022) [78]	Cross-sectional	1499	MeFOSAA, **PFDA**, **PFHxS**, **PFNA**, **PFOA**, **PFOS**, **PFUnDA**	NA	Parity, time since last birth	Plasma	USA

Bold: common across all studies. * Detected in <51% of samples from at least one of the two study sites and thus was excluded from analysis in the study itself. MeFOSAA: N-methylperfluorooctane sulfonamidoacetic acid. PFBA: perfluorobutanoic acid. PFBS: perfluorobutane sulfonate. PFDA: perfluorodecanoic acid. PFDoA: perfluorododecanoic acid. PFHpA: perfluoroheptanoic acid. PFHxA: perfluorohexanoic acid. PFHxS: perfluorohexane sulfonic acid. PFNA: perfluorononanoic acid. PFOA: perfluorooctanoic acid. PFOS: perfluorooctane sulfonic acid; PFPeA: perfluoro-n-pentanoic acid. PFUdA/PFUnA/PFUnDA: perfluoroundecanoic acid.

**Table 4:** Characteristics of preterm birth/gestational age at birth studies.

Study	Study Type	Study Size (n)	Detected PFAS	PFAS Not Detected in 51% or More Samples	Outcomes	Sub-Outcomes	Media	Country
Bangma et al. (2020) [109]	Cross-sectional cohort	122	PFHpS, PFHxS, **PFOS**	6:2 FTS, GenX, PFBA, PFBS, PFDA, PFDoA, PFDoS, PFDS, PFHpA, PFHxA, PFHxDA, PFNA, PFNS, PFOA, PFPeA, PFPeS, PFTeDA, PFTrDA, PFUnA	Gestational age at birth, PTB	Infant sex	Placenta	USA
Chu et al. (2020) [70]	Cohort	372	6:2 Cl-PFESA, PFBA *, PFDA *, PFDoA*, PFHpA *, PFHpS *, PFHxS *, PFNA *, PFOA, **PFOS**, PFUdA *	4:2 FTS, 6:2 FTS, 8:2 Cl-PFESA, 8:2 FTS, FOSA, HFPO-DA, N-EtFOSAA, N-MeFOSAA, PFBS, PFDS, PFHxA, PFNS, PFPeA, PFPeS, PFTeDA, PFTrDA	Gestational age at birth, PTB	Infant sex	Serum	China
Eick and Hom Thepaksorn et al. (2020) [83]	Prospective cohort	506	Me-PFOSA-AcOH, PFDeA *, PFNA, PFHxS, PFOA, **PFOS**, PFUdA *	Et-PFOSA-AcOH, PFBS, PFDoA, PFHpA, PFOSA	Gestational age at birth, PTB	Infant sex	Serum	USA
Hall et al. (2022) [110]	Prospective cohort	120	PFDA, PFNA, PFOA, **PFOS**	GenX, PFBA, PFBS, PFHpA, PFHxA, PFHxS, PFPeA	Gestational age at birth	Infant sex	Placenta	USA
Huo et al. (2020) [68]	Prospective cohort	2849	PFBS, PFDA, PFDoA, PFHpA, PFHxS, PFNA, PFOA, **PFOS**, PFUA	PFOSA	Gestational age at birth, PTB	Spontaneous/non-spontaneous/indicated PTB, spontaneous/indicated late PTB, late PTB (34–36 weeks), length of gestation (weeks); infant sex	Plasma	China
Kalloo et al. (2020) [64]	Prospective cohort	380	PFHxS, PFNA, PFOA, **PFOS**	NA	Gestational age at birth	Infant sex	Serum	USA
Lauritzen et al. (2017) [63]	Case–cohort	424	PFOA, **PFOS**	NA	Gestational age at birth	Infant sex	Serum	Norway
Liu et al. (2020) [84]	Case–control	Cases: 144; Controls: 375	6:2 Cl-PFESA, PFDS, PFHpS, PFHxS, PFOA, **PFOS**	8:2 Cl-PFESA, PFBA, PFBS, PFDA, PFDoA, PFHpA, PFHxA, PFNA, PFPeA, PFTeDA, PFTrDA, PFUdA *	PTB	Spontaneous PTB	Plasma	China
Manzano-Salgado et al. (2017) [74]	Prospective cohort	1202	PFHxS, PFNA, PFOA, **PFOS**	NA	Gestational age at birth, PTB	Infant sex	Plasma	Spain
Meng et al. (2018) [58]	Cohort	3535 (PFOA and PFOS); 2137 (other PFAS)	PFDA, PFHpS, PFHxS, PFNA, PFOA, **PFOS**	NA	Gestational age at birth, PTB	Infant sex	Plasma	Denmark
Sagiv et al.(2017) [59]	Cohort	1645	PFHxS, PFNA, PFOA, **PFOS**	NA	Gestational age at birth, PTB	Infant sex	Plasma	USA
Yang et al. (2022) [108]	Case–control	Cases: 384; Controls: 384	1 m-PFOS, 11Cl-PF3OUdS, 6 m-PFOS, 9Cl-PF3ONS, branched PFHxS, branched PFOS, linear PFHxS, linear PFOS, PFDA, PFDoA, PFHpA, PFHpS, PFHxA, PFNA, PFOA, PFTeDA, PFTrDA, PFUdA, sum 3 + 4 + 5 m-PFOS, sum m2-PFOS, total PFAS, total PFHxS, **total PFOS**	4:2 FTS, 6:2 FTS, 8:2 FTS, FOSA, HFPO-DA, N-EtFOSAA, N-MeFOSAA, PFBA, PFBS, PFDoA, PFDS, PFNS, PFPeA, PFPeS	Gestational age at birth, PTB	Infant sex	Cord serum	China

Bold: common across all studies. * Detected in >51% of samples but not included in the results of original paper, and thus cannot be described here. 11Cl-PF3OUdS: 11-chloroeicosafluoro-3-oxaundecane-1-sulfonic acid. 4:2 FTS: 4:2 Fluorotelomer sulfonic acid. 6:2 Cl-PFESA: 6:2 chlorinated polyfluorinated ether sulfonate. 6:2 FTS: 6:2-fluorotelomersulfonic acid. 8:2 Cl-PFESA: 8:2 chloropolyfluoroether sulfonic acid. 8:2 FTS: 8:2 fluorotelomer sulfonic acid. 9Cl-PF3ONS: perfluoro(2-((6-chlorohexyl)oxy)ethanesulfonic acid). Et-PFOSA-AcOH: 2-(N-ethyl-perfluorooctane sulfonamido) acetic acid. FOSA: perfluorooctane sulfonamide. GenX: Trade name for HFPO-DA (hexafluoropropylene oxide dimer acid). HFPO-DA: hexafluoropropylene oxide dimer acid. Me-PFOSA-AcOH: N-methylperfluorooctane sulfonamidoacetic acid. N-EtFOSAA: N-ethylperfluorooctane sulfonamidoacetic acid. N-MeFOSAA: N-methylperfluorooctane sulfonamidoacetic acid. PFBA: perfluorobutanoic acid. PFBS: perfluorobutane sulfonate. PFDA/PFDeA: perfluorodecanoic acid. PFDoA: perfluorododecanoic acid. PFDOS: perfluorodecane sulfonic acid. PFDS: perfluorodecanesulfonic acid. PFHpA: perfluoroheptanoic acid. PFHpS: perfluoroheptanesulfonic acid. PFHxA: perfluorohexanoic acid. PFHxDA: perfluorohexadecanoic acid. PFHxS: perfluorohexane sulfonate. PFNA: perfluorononanoic acid. PFNS: perfluorononanesulfonic acid. PFOA: perfluorooctanoic acid. PFOS: perfluorooctane sulfonate. PFOSA: perfluorooctane sulfonamide. PFPeA: perfluoro-n-pentanoic acid. PFPeS: perfluoropentanesulfonic acid. PFTeDA: perfluorotetradecanoic acid. PFTrDA: perfluorotridecanoic acid. PFUA/PFUdA/PFUnA: perfluoroundecanoic acid.

**Table 5:** Characteristics of miscarriage studies.

Study	Study Type	Study Size (n)	Detected PFAS	PFAS Not Detected in 51% or More Samples	Outcomes	Media	Country
Liew et al. (2020) [60]	Case–control	Cases: 220; Controls: 218	**PFDA**, PFHpS, **PFHxS**, **PFNA**, **PFOA**, **PFOS**, PFOSA	NA	Miscarriage (12–22 w; second trimester)	Plasma	Denmark
Wang et al. (2021) [71]	Prospective cohort	305 (Site 1: 178; Site 2: 127)	PFBA, PFBS, **PFDA**, PFHxA, **PFNA**, **PFOA**, **PFOS**, **PFHxS**	PFDoA *, PFHpA, PFPeA *, PFUdA *	Preclinical spontaneous abortion (6 w)	Serum	China
Wikström et al. (2021) [76]	Case–control	Cases: 78; Controls: 1449	**PFDA**, PFHpA, **PFHxS**, **PFNA**, **PFOA**, **PFOS**, PFUnDA	NA	Sporadic first trimester miscarriage (≤12 w + 6 d)	Serum	Sweden

Bold: common across all studies. * Detected in <51% of samples from at least one of the two study sites, and thus was excluded from analysis in the study itself. PFBA: perfluorobutanoic acid. PFBS: perfluorobutane sulfonate. PFDA: perfluorodecanoic acid. PFDoA: perfluorododecanoic acid. PFHpA: perfluoroheptanoic acid. PFHpS: perfluoroheptanesulfonic acid. PFHxA: perfluorohexanoic acid. PFHxS: perfluorohexane sulfonate. PFNA: perfluorononanoic acid. PFOA: perfluorooctanoic acid. PFOS: perfluorooctane sulfonate. PFOSA: perfluorooctane sulfonamide. PFPeA: perfluoro-n-pentanoic acid. PFUdA/PFUnDA: perfluoroundecanoic acid.

**Table 8:** Characteristics of sperm health studies.

Study	Study Type	Study Size (n)	Detected PFAS	PFAS Not Detected in 51% or More Samples	Outcomes	Sub-Outcomes	Media	Country
Pan et al. (2019) [69]	Cross-sectional	664	6:2 Cl-PFESA, PFDA, PFNA, **PFOA**, **PFOS**, PFUnDA	Semen: PFHpA, PFDoDA, PFTeDA, PFBS, PFHxS, 8:2 Cl-PFESA; Serum: PFHpA, PFTeDA; (PFTriDA >LOQ in most samples regardless of media, but not included in analysis)	DNA instability, semen volume, sperm concentration, sperm morphology, sperm motility, total sperm count	DNA fragmentation index (%), high DNA stainability %, semen volume (mL), sperm concentration (million/mL), morphologically normal sperm (%), progressive motile sperm (%), straight linear sperm velocity (um/s), curvilinear sperm velocity (um/s), sperm count (million)	Serum, semen	China
Petersen et al. (2018) [61]	Cross-sectional	263	**PFOA**, **PFOS**	NA (PFNA, PFHxS, PFDA >LOQ but not included in analysis)	Semen volume, sperm concentration, sperm morphology, sperm motility, total sperm count	Semen volume (mL), sperm concentration (mill/mL), normal morphology (%), motile sperm (%), total sperm count (million)	Serum, semen	Faroe Islands
Song et al. (2018) [67]	Cross-sectional	103	PFBS, PFBA, PFHpA, PFHS, PFHxA, **PFOA**, **PFOS**, PFPeA, PFPrA, total PFAAs	NA	Sperm concentration, sperm motility	Semen concentration, progressive motility of semen	Blood, semen	China

Bold: common across all studies. 6:2 Cl-PFESA: 6:2 chlorinated polyfluorinated ether sulfonate. 8:2 Cl-PFESA: 8:2 chloropolyfluoroether sulfonic acid. PFAAs: perfluoroalkyl acids. PFBA: perfluorobutanoic acid. PFBS: perfluorobutane sulfonate. PFDA: perfluorodecanoic acid. PFDoDA: perfluorododecanoic acid. PFHpA: perfluoroheptanoic acid. PFHxA: perfluorohexanoic acid. PFHxS/PFHS: perfluorohexane sulfonate. PFNA: perfluorononanoic acid. PFOA: perfluorooctanoic acid. PFOS: perfluorooctane sulfonate. PFPeA: perfluoro-n-pentanoic acid. PFPrA: perfluoropropionic acid. PFTeDA: perfluorotetradecanoic acid. PFTriDA: perfluorotridecanoic acid. PFUnDA: perfluoroundecanoic acid.

**Table 9:** Characteristics of fetal growth studies.

Study	Study Type	Study Size (n)	Detected PFAS	PFAS Not Detected in 51% or More Samples	Outcomes	Sub-Outcomes	Media	Country
Costa et al. (2019) [73]	Cohort	1230	**PFHxS**, **PFNA**, **PFOA**, **PFOS**	NA	Cranium size, appendicular skeleton length, abdominal circumference	Biparietal diameter, femur length, estimated fetal weight, abdominal circumference	Plasma	USA
Ouidir et al. (2020) [77]	Cohort	2284	N-MeFOSAA, PFDA, PFDoDA, **PFHxS**, **PFNA**, **PFOA**, **PFOS**, PFUnDA	PFDS, PFHpA PFOSA	Cranium size, appendicular skeleton length, abdominal circumference	Cerebral width, head circumference, inner orbit diameter, occipital–frontal diameter, outer orbit diameter, biparietal diameter, fibula length, humerus length, radial length, tibia length, ulnar length, foot length, femur length, estimated fetal growth, abdominal circumference	Plasma	Spain

Bold: common across all studies. N-MeFOSAA: N-methylperfluorooctane sulfonamidoacetic acid. PFDA: perfluorodecanoic acid. PFDoDA: perfluorododecanoic acid. PFDS: perfluorodecanesulfonic acid. PFHpA: perfluoroheptanoic acid. PFHxS: perfluorohexane sulfonate. PFNA: perfluorononanoic acid. PFOA: perfluorooctanoic acid. PFOS: perfluorooctane sulfonate. PFOSA: perfluorooctane sulfonamide.

**Table 10:** Summary of conclusions.

Endpoint	Conclusions
Fertility	Clinical pregnancy and infertility were not affected by PFAS.
Preterm birth (PTB)/gestational age at birth (GAB)	In roughly one-third of all PTB/GAB studies, a decrease in gestational age at birth was inversely associated with PFOS levels, though the 1–3-day earlier birth is likely clinically irrelevant for preterm birth, defined as <37 weeks of gestation.
Fetal growth	Fetal growth outcomes were inconsistent, though the combination of PFAS and smoking may play an unconfirmed role in reducing growth.
Miscarriage	In two of the three miscarriage studies, PFOA was associated with weakly to moderately increased odds of miscarriage, especially in parous women.
Menstruation/menopause	Menstrual cycle length and regularity were unaffected, though heavy bleeding was reduced with nearly every PFAS studied. In a single study, increasing tertiles of PFOA and PFOS affected menopause approximately one year earlier than the lowest exposure group, and high total PFAS levels predicted two years earlier. PFOA and PFOS were associated with up to six times higher odds for primary ovarian insufficiency.
Ovarian health	Lesser studied PFAS such as PFDoDA and PFBS were implicated in increasing odds of PCOS- and endometriosis-related infertility in women who seek IVF, though this may be due to an unknown lifestyle factor.
Sperm health	Sperm motility was decreased with nearly every PFAS when measured in semen but not serum; similarly, most PFASs (except PFOA and PFOS) affected DNA stability in semen and serum.

PFOS: perfluorooctane sulfonic acid. PFOA: perfluoroctanoic acid, PFDoDA: perfluorododecanoic acid. PFBS: perfluorobutanesulfonic acid.

## Data Availability

Data are contained within the article and Supplementary Materials.

## Supplementary Materials

The following supporting information can be downloaded at: https://www.mdpi.com/article/10.3390/ijerph21121615/s1, File S1: Newcastle–Ottawa Scale adapted rubrics for risk of bias scoring. Tables S1 and S2: Risk of bias scores for each report.

## Registration and Protocol

This review and protocol were not registered. No separate protocol from the methods section was prepared.

## References

[1] Roseboom T.J., van der Meulen J.H.P., Ravelli A.C.J., Osmond C., Barker D.J.P., Bleker O.P. (2001). Effects of Prenatal Exposure to the Dutch Famine on Adult Disease in Later Life: An Overview. Twin Res..

[2] Laine J.E., Ray P., Bodnar W., Cable P.H., Boggess K., Offenbacher S., Fry R.C. (2015). Placental Cadmium Levels Are Associated with Increased Preeclampsia Risk. PLoS ONE.

[3] Toichuev R.M., Zhilova L.V., Paizildaev T.R., Khametova M.S., Rakhmatillaev A., Sakibaev K.S., Madykova Z.A., Toichueva A.U., Schlumpf M., Weber R. (2017). Organochlorine Pesticides in Placenta in Kyrgyzstan and the Effect on Pregnancy, Childbirth, and Newborn Health. Environ. Sci. Pollut. Res..

[4] Leclerc F., Dubois M.-F., Aris A. (2014). Maternal, Placental and Fetal Exposure to Bisphenol a in Women with and without Preeclampsia. Hypertens. Pregnancy.

[5] Li J., Cai D., Chu C., Li Q., Zhou Y., Hu L., Yang B.-Y., Dong G.-H., Zeng X.-W., Chen D. (2020). Transplacental Transfer of Per- and Polyfluoroalkyl Substances (PFASs): Differences between Preterm and Full-Term Deliveries and Associations with Placental Transporter MRNA Expression. Environ. Sci. Technol..

[6] Mamsen L.S., Björvang R.D., Mucs D., Vinnars M.-T., Papadogiannakis N., Lindh C.H., Andersen C.Y., Damdimopoulou P. (2019). Concentrations of Perfluoroalkyl Substances (PFASs) in Human Embryonic and Fetal Organs from First, Second, and Third Trimester Pregnancies. Environ. Int..

[7] Ackerman Grunfeld D., Gilbert D., Hou J., Jones A.M., Lee M.J., Kibbey T.C.G., O’Carroll D.M. (2024). Underestimated Burden of Per- and Polyfluoroalkyl Substances in Global Surface Waters and Groundwaters. Nat. Geosci..

[8] Kuo K.-Y., Chen Y., Chuang Y., Lin P., Lin Y.-J. (2023). Worldwide Serum Concentration-Based Probabilistic Mixture Risk Assessment of Perfluoroalkyl Substances among Pregnant Women, Infants, and Children. Ecotoxicol. Environ. Saf..

[9] Muir D., Bossi R., Carlsson P., Evans M., De Silva A., Halsall C., Rauert C., Herzke D., Hung H., Letcher R. (2019). Levels and Trends of Poly- and Perfluoroalkyl Substances in the Arctic Environment—An Update. Emerg. Contam..

[10] Young C.J., Furdui V.I., Franklin J., Koerner R.M., Muir D.C.G., Mabury S.A. (2007). Perfluorinated Acids in Arctic Snow:  New Evidence for Atmospheric Formation. Environ. Sci. Technol..

[11] Miranda D.d.A., Leonel J., Benskin J.P., Johansson J., Hatje V. (2021). Perfluoroalkyl Substances in the Western Tropical Atlantic Ocean. Environ. Sci. Technol..

[12] O’Hagan D. (2008). Understanding Organofluorine Chemistry. An Introduction to the C–F Bond. Chem. Soc. Rev..

[13] Gaines L.G.T. (2022). Historical and Current Usage of Per- and Polyfluoroalkyl Substances (PFAS): A Literature Review. Am. J. Ind. Med..

[14] Prevedouros K., Cousins I.T., Buck R.C., Korzeniowski S.H. (2006). Sources, Fate and Transport of Perfluorocarboxylates. Environ. Sci. Technol..

[15] Fenton S.E., Ducatman A., Boobis A., DeWitt J.C., Lau C., Ng C., Smith J.S., Roberts S.M. (2020). Per- and Polyfluoroalkyl Substance Toxicity and Human Health Review: Current State of Knowledge and Strategies for Informing Future Research. Environ. Toxicol. Chem..

[16] Calafat A.M., Kuklenyik Z., Reidy J.A., Caudill S.P., Tully J.S., Needham L.L. (2007). Serum Concentrations of 11 Polyfluoroalkyl Compounds in the U.S. Population: Data from the National Health and Nutrition Examination Survey (NHANES) 1999−2000. Environ. Sci. Technol..

[17] Centers for Disease Control and Prevention National Health and Nutrition Examination Survey Data: 2017–2018 Data Documentation, Codebook, and Frequencies: Perfluoroalkyl and Polyfluoroalkyl Substances. https://wwwn.cdc.gov/Nchs/Nhanes/2017-2018/PFAS_J.htm#LBXNFOS.

[18] Olsen G.W., Burris J.M., Ehresman D.J., Froehlich J.W., Seacat A.M., Butenhoff J.L., Zobel L.R. (2007). Half-Life of Serum Elimination of Perfluorooctanesulfonate, Perfluorohexanesulfonate, and Perfluorooctanoate in Retired Fluorochemical Production Workers. Environ. Health Perspect..

[19] He Y., Lv D., Li C., Liu X., Liu W., Han W. (2022). Human Exposure to F-53B in China and the Evaluation of Its Potential Toxicity: An Overview. Environ. Int..

[20] Schultz A.A., Stanton N., Shelton B., Pomazal R., Lange M.A., Irving R., Meiman J., Malecki K.C. (2023). Biomonitoring of Perfluoroalkyl and Polyfluoroalkyl Substances (PFAS) from the Survey of the Health of Wisconsin (SHOW) 2014–2016 and Comparison with the National Health and Nutrition Examination Survey (NHANES). J. Expo. Sci. Environ. Epidemiol..

[21] Shi Y., Vestergren R., Xu L., Zhou Z., Li C., Liang Y., Cai Y. (2016). Human Exposure and Elimination Kinetics of Chlorinated Polyfluoroalkyl Ether Sulfonic Acids (Cl-PFESAs). Environ. Sci. Technol..

[22] United States Environmental Protection Agency Drinking Water Health Advisory for Perfluorooctanoic Acid (PFOA), Last Revised May 2016. https://www.epa.gov/sites/default/files/2016-05/documents/pfoa_health_advisory_final_508.pdf.

[23] Rand A.A., Mabury S.A. (2017). Is There a Human Health Risk Associated with Indirect Exposure to Perfluoroalkyl Carboxylates (PFCAs)?. Toxicology.

[24] Darrow L.A., Stein C.R., Steenland K. (2013). Serum Perfluorooctanoic Acid and Perfluorooctane Sulfonate Concentrations in Relation to Birth Outcomes in the Mid-Ohio Valley, 2005–2010. Environ. Health Perspect..

[25] Riise H.K.R., Sulo G., Tell G.S., Igland J., Nygård O., Iversen A., Daltveit A.K. (2018). Association between Gestational Hypertension and Risk of Cardiovascular Disease among 617 589 Norwegian Women. J. Am. Heart Assoc..

[26] Davis E.F., Lewandowski A.J., Aye C., Williamson W., Boardman H., Huang R.-C., Mori T.A., Newnham J., Beilin L.J., Leeson P. (2015). Clinical Cardiovascular Risk during Young Adulthood in Offspring of Hypertensive Pregnancies: Insights from a 20-Year Prospective Follow-up Birth Cohort. BMJ Open.

[27] Timpka S., Macdonald-Wallis C., Hughes A.D., Chaturvedi N., Franks P.W., Lawlor D.A., Fraser A. (2016). Hypertensive Disorders of Pregnancy and Offspring Cardiac Structure and Function in Adolescence. J. Am. Heart Assoc..

[28] Timmermann A., Avenbuan O.N., Romano M.E., Braun J.M., Tolstrup J.S., Vandenberg L.N., Fenton S.E. (2023). Per- and Polyfluoroalkyl Substances and Breastfeeding as a Vulnerable Function: A Systematic Review of Epidemiological Studies. Toxics.

[29] Rickard B.P., Rizvi I., Fenton S.E. (2022). Per- and Poly-Fluoroalkyl Substances (PFAS) and Female Reproductive Outcomes: PFAS Elimination, Endocrine-Mediated Effects, and Disease. Toxicology.

[30] Vestergaard S., Nielsen F., Andersson A.-M., Hjollund N.H., Grandjean P., Andersen H.R., Jensen T.K. (2012). Association between Perfluorinated Compounds and Time to Pregnancy in a Prospective Cohort of Danish Couples Attempting to Conceive. Hum. Reprod..

[31] Fei C., McLaughlin J.K., Lipworth L., Olsen J. (2009). Maternal Levels of Perfluorinated Chemicals and Subfecundity. Hum. Reprod..

[32] Bach C.C., Liew Z., Bech B.H., Nohr E.A., Fei C., Bonefeld-Jorgensen E.C., Henriksen T.B., Olsen J. (2015). Perfluoroalkyl Acids and Time to Pregnancy Revisited: An Update from the Danish National Birth Cohort. Environ. Health.

[33] Kahn L.G., Harley K.G., Siegel E.L., Zhu Y., Factor-Litvak P., Porucznik C.A., Klein-Fedyshin M., Hipwell A.E., Program Collaborators for Environmental Influences on Child Health Outcomes Program (2021). Persistent organic pollutants and couple fecundability: A systematic review. Hum. Reprod. Update.

[34] Wang W., Hong X., Zhao F., Wu J., Wang B. (2023). The Effects of Perfluoroalkyl and Polyfluoroalkyl Substances on Female Fertility: A Systematic Review and Meta-Analysis. Environ. Res..

[35] Calvert L., Green M.P., De Iuliis G.N., Dun M.D., Turner B.D., Clarke B.O., Eamens A.L., Roman S.D., Nixon B. (2022). Assessment of the Emerging Threat Posed by Perfluoroalkyl and Polyfluoroalkyl Substances to Male Reproduction in Humans. Front. Endocrinol..

[36] Tarapore P., Ouyang B. (2021). Perfluoroalkyl Chemicals and Male Reproductive Health: Do PFOA and PFOS Increase Risk for Male Infertility?. Int. J. Environ. Res. Public Health.

[37] Sun Z., Wen Y., Wang B., Deng S., Zhang F., Fu Z., Yuan Y., Zhang D. (2023). Toxic Effects of Per- and Polyfluoroalkyl Substances on Sperm: Epidemiological and Experimental Evidence. Front. Endocrinol..

[38] Guerrero-Bosagna C., Skinner M.K. (2014). Environmentally Induced Epigenetic Transgenerational Inheritance of Male Infertility. Curr. Opin. Genet. Dev..

[39] Fullston T., McPherson N.O., Owens J.A., Kang W.X., Sandeman L.Y., Lane M. (2015). Paternal Obesity Induces Metabolic and Sperm Disturbances in Male Offspring That Are Exacerbated by Their Exposure to an “Obesogenic” Diet. Physiol. Rep..

[40] Barouki R., Melén E., Herceg Z., Beckers J., Chen J., Karagas M., Puga A., Xia Y., Chadwick L., Yan W. (2018). Epigenetics as a Mechanism Linking Developmental Exposures to Long-Term Toxicity. Environ. Int..

[41] Heijmans B.T., Tobi E.W., Stein A.D., Putter H., Blauw G.J., Susser E.S., Slagboom P.E., Lumey L.H. (2008). Persistent Epigenetic Differences Associated with Prenatal Exposure to Famine in Humans. Proc. Natl. Acad. Sci. USA.

[42] Stener-Victorin E., Deng Q. (2021). Epigenetic Inheritance of Polycystic Ovary Syndrome—Challenges and Opportunities for Treatment. Nat. Rev. Endocrinol..

[43] Bhattacharya S., Amalraj Raja E., Ruiz Mirazo E., Campbell D.M., Lee A.J., Norman J.E., Bhattacharya S. (2010). Inherited Predisposition to Spontaneous Preterm Delivery. Obstet. Gynecol..

[44] Vargesson N. (2015). Thalidomide-Induced Teratogenesis: History and Mechanisms. Birth Defects Res. Part C Embryo Today Rev..

[45] Mocarelli P., Gerthoux P.M., Needham L.L., Patterson D.G., Limonta G., Falbo R., Signorini S., Bertona M., Crespi C., Sarto C. (2011). Perinatal Exposure to Low Doses of Dioxin Can Permanently Impair Human Semen Quality. Environ. Health Perspect..

[46] Mocarelli P., Gerthoux P.M., Patterson D.G., Milani S., Limonta G., Bertona M., Signorini S., Tramacere P., Colombo L., Crespi C. (2008). Dioxin Exposure, from Infancy through Puberty, Produces Endocrine Disruption and Affects Human Semen Quality. Environ. Health Perspect..

[47] Hu X.C., Tokranov A.K., Liddie J., Zhang X., Grandjean P., Hart J.E., Laden F., Sun Q., Yeung L.W.Y., Sunderland E.M. (2019). Tap Water Contributions to Plasma Concentrations of Poly- and Perfluoroalkyl Substances (PFAS) in a Nationwide Prospective Cohort of U.S. Women. Environ. Health Perspect..

[48] Andrews D.Q., Naidenko O.V. (2020). Population-Wide Exposure to Per- and Polyfluoroalkyl Substances from Drinking Water in the United States. Environ. Sci. Technol. Lett..

[49] United States Environmental Protection Agency Emerging Contaminants—Perfluorooctane Sulfonate (PFOS) and Perfluorooctanoic Acid (PFOA). EPA 505-F-14-001. Solid Waste and Emergency Responses. https://semspub.epa.gov/work/HQ/100002767.pdf.

[50] Müller C.E., De Silva A.O., Small J., Williamson M., Wang X., Morris A., Katz S., Gamberg M., Muir D.C.G. (2011). Biomagnification of Perfluorinated Compounds in a Remote Terrestrial Food Chain: Lichen–Caribou–Wolf. Environ. Sci. Technol..

[51] Miranda D.A., Zachritz A.M., Whitehead H.D., Cressman S.R., Peaslee G.F., Lamberti G.A. (2023). Occurrence and Biomagnification of Perfluoroalkyl Substances (PFAS) in Lake Michigan Fishes. Sci. Total Environ..

[52] United States Environmental Protection Agency, Office of Water (2023). Drinking Water Infrastructure Needs Survey and Assessment, 7th Report to Congress. https://www.epa.gov/system/files/documents/2023-09/Seventh%20DWINSA_September2023_Final.pdf.

[53] The Economic Benefits of Investing in Water Infrastructure How a Failure to Act Would Affect the US Economic Recovery. https://uswateralliance.org/wp-content/uploads/2023/09/VOW-Economic-Paper_1.pdf.

[54] United States Environmental Protection Agency FACT SHEET Bipartisan Infrastructure Law: State Revolving Funds Implementation Memorandum, last revised March 2022. https://www.epa.gov/system/files/documents/2022-03/bil-srf-memo-fact-sheet-final.pdf.

[55] Cordner A., De La Rosa V.Y., Schaider L.A., Rudel R.A., Richter L., Brown P. (2019). Guideline Levels for PFOA and PFOS in Drinking Water: The Role of Scientific Uncertainty, Risk Assessment Decisions, and Social Factors. J. Expo. Sci. Environ. Epidemiol..

[56] Howard B.E., Phillips J., Tandon A., Maharana A., Elmore R., Mav D., Sedykh A., Thayer K., Merrick B.A., Walker V. (2020). SWIFT-Active Screener: Accelerated Document Screening through Active Learning and Integrated Recall Estimation. Environ. Int..

[57] Page M.J., McKenzie J.E., Bossuyt P.M., Boutron I., Hoffmann T.C., Mulrow C.D., Shamseer L., Tetzlaff J.M., Akl E.A., Brennan S.E. (2021). The PRISMA 2020 statement: An Updated Guideline for Reporting Systematic Reviews. BMJ.

[58] Meng Q., Inoue K., Ritz B., Olsen J., Liew Z. (2018). Prenatal Exposure to Perfluoroalkyl Substances and Birth Outcomes; An Updated Analysis from the Danish National Birth Cohort. Int. J. Environ. Res. Public Health.

[59] Sagiv S.K., Rifas-Shiman S.L., Fleisch A.F., Webster T.F., Calafat A.M., Ye X., Gillman M.W., Oken E. (2017). Early-Pregnancy Plasma Concentrations of Perfluoroalkyl Substances and Birth Outcomes in Project Viva: Confounded by Pregnancy Hemodynamics?. Am. J. Epidemiol..

[60] Liew Z., Luo J., Nohr E.A., Bech B.H., Bossi R., Arah O.A., Olsen J. (2020). Maternal Plasma Perfluoroalkyl Substances and Miscarriage: A Nested Case–Control Study in the Danish National Birth Cohort. Environ. Health Perspect..

[61] Petersen M., Halling J., Jørgensen N., Nielsen F., Grandjean P., Jensen T., Weihe P. (2018). Reproductive Function in a Population of Young Faroese Men with Elevated Exposure to Polychlorinated Biphenyls (PCBs) and Perfluorinated Alkylate Substances (PFAS). Int. J. Environ. Res. Public Health.

[62] Ding N., Harlow S.D., Randolph J.F., Calafat A.M., Mukherjee B., Batterman S., Gold E.B., Park S.K. (2020). Associations of Perfluoroalkyl Substances with Incident Natural Menopause: The Study of Women’s Health across the Nation. J. Clin. Endocrinol. Metab..

[63] Lauritzen H.B., Larose T.L., Øien T., Sandanger T.M., Odland J.Ø., van de Bor M., Jacobsen G.W. (2016). Maternal Serum Levels of Perfluoroalkyl Substances and Organochlorines and Indices of Fetal Growth: A Scandinavian Case–Cohort Study. Pediatr. Res..

[64] Kalloo G., Wellenius G.A., McCandless L.C., Calafat A.M., Sjödin A., Romano M.E., Karagas M.R., Chen A., Yolton K., Lanphear B.P. (2020). Exposures to Chemical Mixtures during Pregnancy and Neonatal Outcomes: The HOME Study. Environ. Int..

[65] Singer A.B., Whitworth K.W., Haug L.S., Sabaredzovic A., Impinen A., Papadopoulou E., Longnecker M.P. (2018). Menstrual Cycle Characteristics as Determinants of Plasma Concentrations of Perfluoroalkyl Substances (PFASs) in the Norwegian Mother and Child Cohort (MoBa Study). Environ. Res..

[66] Zhou W., Zhang L., Tong C., Fang F., Zhao S., Tian Y., Tao Y., Zhang J. (2017). Plasma Perfluoroalkyl and Polyfluoroalkyl Substances Concentration and Menstrual Cycle Characteristics in Preconception Women. Environ. Health Perspect..

[67] Song X., Tang S., Zhu H., Chen Z., Zang Z., Zhang Y., Niu X., Wang X., Yin H., Zeng F. (2018). Biomonitoring PFAAs in Blood and Semen Samples: Investigation of a Potential Link between PFAAs Exposure and Semen Mobility in China. Environ. Int..

[68] Huo X., Zhang L., Huang R., Feng L., Wang W., Zhang J. (2020). Perfluoroalkyl Substances Exposure in Early Pregnancy and Preterm Birth in Singleton Pregnancies: A Prospective Cohort Study. Environ. Health.

[69] Pan Y., Cui Q., Wang J., Sheng N., Jing J., Yao B., Dai J. (2019). Profiles of Emerging and Legacy Per-/Polyfluoroalkyl Substances in Matched Serum and Semen Samples: New Implications for Human Semen Quality. Environ. Health Perspect..

[70] Chu C., Zhou Y., Li Q.-Q., Bloom M.S., Lin S., Yu Y.-J., Chen D., Yu H.-Y., Hu L.-W., Yang B.-Y. (2020). Are Perfluorooctane Sulfonate Alternatives Safer? New Insights from a Birth Cohort Study. Environ. Int..

[71] Wang B., Fu J., Gao K., Liu Q., Zhuang L., Zhang G., Long M., Na J., Ren M., Wang A. (2021). Early Pregnancy Loss: Do Per- and Polyfluoroalkyl Substances Matter?. Environ. Int..

[72] Wang B., Zhang R., Jin F., Lou H., Mao Y., Zhu W., Zhou W., Zhang P., Zhang J. (2017). Perfluoroalkyl Substances and Endometriosis-Related Infertility in Chinese Women. Environ. Int..

[73] Costa O., Iñiguez C., Manzano-Salgado C.B., Amiano P., Murcia M., Casas M., Irizar A., Basterrechea M., Beneito A., Schettgen T. (2019). First-Trimester Maternal Concentrations of Polyfluoroalkyl Substances and Fetal Growth throughout Pregnancy. Environ. Int..

[74] Manzano-Salgado C.B., Casas M., Lopez-Espinosa M.-J., Ballester F., Iñiguez C., Martinez D., Costa O., Santa-Marina L., Pereda-Pereda E., Schettgen T. (2017). Prenatal Exposure to Perfluoroalkyl Substances and Birth Outcomes in a Spanish Birth Cohort. Environ. Int..

[75] Zhang S., Tan R., Pan R., Xiong J., Tian Y., Wu J., Chen L. (2018). Association of Perfluoroalkyl and Polyfluoroalkyl Substances with Premature Ovarian Insufficiency in Chinese Women. J. Clin. Endocrinol. Metab..

[76] Wikström S., Hussein G., Lingroth Karlsson A., Lindh C.H., Bornehag C.-G. (2021). Exposure to Perfluoroalkyl Substances in Early Pregnancy and Risk of Sporadic First Trimester Miscarriage. Sci. Rep..

[77] Ouidir M., Buck Louis G.M., Kanner J., Grantz K.L., Zhang C., Sundaram R., Rahman M.L., Lee S., Kannan K., Tekola-Ayele F. (2020). Association of Maternal Exposure to Persistent Organic Pollutants in Early Pregnancy with Fetal Growth. JAMA Pediatr..

[78] Wise L.A., Wesselink A.K., Schildroth S., Calafat A.M., Bethea T.N., Geller R.J., Coleman C.M., Fruh V., Henn B.G., Botelho J.C. (2022). Correlates of Plasma Concentrations of Per- and Poly-Fluoroalkyl Substances among Reproductive-Aged Black Women. Environ. Res..

[79] March of Dimes A Profile of Prematurity of United States. https://www.marchofdimes.org/peristats/reports/united-states/prematurity-profile.

[80] Björvang R.D., Hallberg I., Pikki A., Berglund L., Pedrelli M., Kiviranta H., Rantakokko P., Ruokojärvi P., Lindh C.H., Olovsson M. (2022). Follicular Fluid and Blood Levels of Persistent Organic Pollutants and Reproductive Outcomes among Women Undergoing Assisted Reproductive Technologies. Environ. Res..

[81] Heffernan A., Cunningham T.J., Drage D.S., Aylward L.L., Thompson K.L., Vijayasarathy S., Mueller J., Atkin S.L., Sathyapalan T. (2018). Perfluorinated Alkyl Acids in the Serum and Follicular Fluid of UK Women with and without Polycystic Ovarian Syndrome Undergoing Fertility Treatment and Associations with Hormonal and Metabolic Parameters. Int. J. Hyg. Environ. Health.

[82] Björvang R.D., Hassan J., Stefopoulou M., Gemzell-Danielsson K., Pedrelli M., Kiviranta H., Rantakokko P., Ruokojärvi P., Lindh C.H., Acharya G. (2021). Persistent Organic Pollutants and the Size of Ovarian Reserve in Reproductive-Aged Women. Environ. Int..

[83] Eick S.M., Hom Thepaksorn E.K., Izano M., Cushing L., Wang Y., Smith S.C., Gao S., Park J.-S., Padula A., DeMicco E. (2020). Associations between Prenatal Maternal Exposure to Per- and Polyfluoroalkyl Substances (PFAS) and Polybrominated Diphenyl Ethers (PBDEs) and Birth Outcomes among Pregnant Women in San Francisco. Environ. Health.

[84] Liu X., Chen D., Wang B., Xu F., Pang Y., Zhang L., Zhang Y., Jin L., Li Z., Ren A. (2020). Does Low Maternal Exposure to Per- and Polyfluoroalkyl Substances Elevate the Risk of Spontaneous Preterm Birth? A Nested Case–Control Study in China. Environ. Sci. Technol..

[85] Herzog R., Álvarez-Pasquin M.J., Díaz C., Del Barrio J.L., Estrada J.M., Gil Á. (2013). Are Healthcare Workers’ Intentions to Vaccinate Related to Their Knowledge, Beliefs and Attitudes? A Systematic Review. BMC Public Health.

[86] Wells G., Shea B., O’Connell D., Peterson J., Welch V., Losos M., Tugwell P. The Newcastle-Ottawa Scale (NOS) for Assessing the Quality of Nonrandomised Studies in Meta-Analyses. https://www.ohri.ca/programs/clinical_epidemiology/oxford.asp.

[87] The BMJ Correlation and Regression. https://www.bmj.com/about-bmj/resources-readers/publications/statistics-square-one/11-correlation-and-regression.

[88] Cohen J. (1988). Statistical Power Analysis for the Behavioral Sciences.

[89] Rosenthal J.A. (1996). Qualitative Descriptors of Strength of Association and Effect Size. J. Soc. Serv. Res..

[90] Carson S.A., Kallen A.N. (2021). Diagnosis and Management of Infertility: A Review. JAMA.

[91] Bach C.C., Vested A., Jørgensen K.T., Bonde J.P.E., Henriksen T.B., Toft G. (2016). Perfluoroalkyl and Polyfluoroalkyl Substances and Measures of Human Fertility: A Systematic Review. Crit. Rev. Toxicol..

[92] Holte J., Berglund L., Milton K., Garello C., Gennarelli G., Revelli A., Bergh T. (2006). Construction of an Evidence-Based Integrated Morphology Cleavage Embryo Score for Implantation Potential of Embryos Scored and Transferred on Day 2 after Oocyte Retrieval. Hum. Reprod..

[93] Jirge P. (2011). Ovarian Reserve Tests. J. Hum. Reprod. Sci..

[94] Olsen G.W., Butenhoff J.L., Zobel L.R. (2009). Perfluoroalkyl Chemicals and Human Fetal Development: An Epidemiologic Review with Clinical and Toxicological Perspectives. Reprod. Toxicol..

[95] Appel M., Forsthuber M., Ramos R., Widhalm R., Granitzer S., Uhl M., Hengstschläger M., Stamm T., Gundacker C. (2021). The Transplacental Transfer Efficiency of Per- and Polyfluoroalkyl Substances (PFAS): A First Meta-Analysis. J. Toxicol. Environ. Health Part B.

[96] Baird D.D., Harmon Q.E., Upson K., Moore K.R., Barker-Cummings C., Baker S., Cooper T., Wegienka G. (2015). A Prospective, Ultrasound-Based Study to Evaluate Risk Factors for Uterine Fibroid Incidence and Growth: Methods and Results of Recruitment. J. Women’s Health.

[97] Green M.P., Harvey A.J., Finger B.J., Tarulli G.A. (2021). Endocrine Disrupting Chemicals: Impacts on Human Fertility and Fecundity during the Peri-Conception Period. Environ. Res..

[98] Waitzman N.J., Jalali A., Grosse S.D. (2021). Preterm Birth Lifetime Costs in the United States in 2016: An Update. Semin. Perinatol..

[99] Beam A.L., Fried I., Palmer N., Agniel D., Brat G., Fox K., Kohane I., Sinaiko A., Zupancic J.A.F., Armstrong J. (2020). Estimates of Healthcare Spending for Preterm and Low-Birthweight Infants in a Commercially Insured Population: 2008–2016. Obstet. Gynecol. Surv..

[100] Pravia C., Benny M. (2020). Long-Term Consequences of Prematurity. Clevel. Clin. J. Med..

[101] Stock S.J., Bauld L. (2020). Maternal Smoking and Preterm Birth: An Unresolved Health Challenge. PLoS Med..

[102] Albertsen K. (2004). Alcohol Consumption during Pregnancy and the Risk of Preterm Delivery. Am. J. Epidemiol..

[103] Ikehara S., Kimura T., Kakigano A., Sato T., Iso H., Saito H., Kishi R., Yaegashi N., Hashimoto K., Mori C. (2019). Association between Maternal Alcohol Consumption during Pregnancy and Risk of Preterm Delivery: The Japan Environment and Children’s Study. BJOG Int. J. Obstet. Gynaecol..

[104] Stone J., Sutrave P., Gascoigne E., Givens M.B., Fry R.C., Manuck T.A. (2021). Exposure to Toxic Metals and Per- and Polyfluoroalkyl Substances and the Risk of Preeclampsia and Preterm Birth in the United States: A Review. Am. J. Obstet. Gynecol. MFM.

[105] Gao X., Ni W., Zhu S., Wu Y., Cui Y., Ma J., Liu Y., Qiao J., Ye Y., Yang P. (2021). Per- and Polyfluoroalkyl Substances Exposure during Pregnancy and Adverse Pregnancy and Birth Outcomes: A Systematic Review and Meta-Analysis. Environ. Res..

[106] Deji Z., Liu P., Wang X., Zhang X., Luo Y., Huang Z. (2021). Association between Maternal Exposure to Perfluoroalkyl and Polyfluoroalkyl Substances and Risks of Adverse Pregnancy Outcomes: A Systematic Review and Meta-Analysis. Sci. Total Environ..

[107] Gui S.-Y., Chen Y.-N., Wu K.-J., Liu W., Wang W.-J., Liang H.-R., Jiang Z.-X., Li Z.-L., Hu C.-Y. (2022). Association between Exposure to Per- and Polyfluoroalkyl Substances and Birth Outcomes: A Systematic Review and Meta-Analysis. Front. Public Health.

[108] Yang B.-Y., Wu J., Niu X., He C., Bloom M.S., Abudoukade M., Abulizi M., Xu A., Li B., Li L. (2022). Low-Level Environmental Per- and Polyfluoroalkyl Substances and Preterm Birth: A Nested Case–Control Study among a Uyghur Population in Northwestern China. Expo. Health.

[109] Bangma J., Eaves L.A., Oldenburg K., Reiner J.L., Manuck T., Fry R.C. (2020). Identifying Risk Factors for Levels of Per- and Polyfluoroalkyl Substances (PFAS) in the Placenta in a High-Risk Pregnancy Cohort in North Carolina. Environ. Sci. Technol..

[110] Hall S.M., Zhang S., Hoffman K., Miranda M.L., Stapleton H.M. (2022). Concentrations of Per- and Polyfluoroalkyl Substances (PFAS) in Human Placental Tissues and Associations with Birth Outcomes. Chemosphere.

[111] Mokra K. (2021). Endocrine Disruptor Potential of Short- and Long-Chain Perfluoroalkyl Substances (PFASs)—A Synthesis of Current Knowledge with Proposal of Molecular Mechanism. Int. J. Mol. Sci..

[112] Kjeldsen L.S., Bonefeld-Jørgensen E.C. (2013). Perfluorinated Compounds Affect the Function of Sex Hormone Receptors. Environ. Sci. Pollut. Res..

[113] Rivera-Núñez Z., Kinkade C.W., Khoury L., Brunner J., Murphy H., Wang C., Kannan K., Miller R.K., O’Connor T.G., Barrett E.S. (2023). Prenatal Perfluoroalkyl Substances Exposure and Maternal Sex Steroid Hormones across Pregnancy. Environ. Res..

[114] Xie X., Weng X., Liu S., Chen J., Guo X., Gao X., Fei Q., Hao G., Jing C., Feng L. (2021). Perfluoroalkyl and Polyfluoroalkyl Substance Exposure and Association with Sex Hormone Concentrations: Results from the NHANES 2015–2016. Environ. Sci. Eur..

[115] Pavan A., Cendron L., Di Nisio A., Pedrucci F., Sabovic I., Scarso A., Ferlin A., Angelini A., Foresta C., De Toni L. (2023). In Vitro Binding Analysis of Legacy-Linear and New Generation-Cyclic Perfluoro-Alkyl Substances on Sex Hormone Binding Globulin and Albumin, Suggests Low Impact on Serum Hormone Kinetics of Testosterone. Toxicology.

[116] (2023). National Center for Environmental Health. National Report on Human Exposure to Environmental Chemicals. Updated March 2024. https://stacks.cdc.gov/view/cdc/133100.

[117] Wang Q., Ruan Y., Jin L., Tao L.S., Lai H., Li G., Yeung L.W., Leung K.M., Lam P.K. (2023). Legacy and Emerging Per- and Polyfluoroalkyl Substances in a Subtropical Marine Food Web: Suspect Screening, Isomer Profile, and Identification of Analytical Interference. Environ. Sci. Technol..

[118] Goldenberg R.L., Gravett M.G., Iams J., Papageorghiou A.T., Waller S.A., Kramer M., Culhane J., Barros F., Conde-Agudelo A., Bhutta Z.A. (2012). The Preterm Birth Syndrome: Issues to Consider in Creating a Classification System. Am. J. Obstet. Gynecol..

[119] Kramer M.S., Papageorghiou A., Culhane J., Bhutta Z., Goldenberg R.L., Gravett M., Iams J.D., Conde-Agudelo A., Waller S., Barros F. (2012). Challenges in Defining and Classifying the Preterm Birth Syndrome. Am. J. Obstet. Gynecol..

[120] Quenby S., Gallos I.D., Dhillon-Smith R.K., Podesek M., Stephenson M.D., Fisher J., Brosens J.J., Brewin J., Ramhorst R., Lucas E.S. (2021). Miscarriage Matters: The Epidemiological, Physical, Psychological, and Economic Costs of Early Pregnancy Loss. Lancet.

[121] Wang Y.-X., Mínguez-Alarcón L., Gaskins A.J., Missmer S.A., Rich-Edwards J.W., Manson J.E., Pan A., Chavarro J.E. (2021). Association of Spontaneous Abortion with All Cause and Cause Specific Premature Mortality: Prospective Cohort Study. BMJ.

[122] Harty T., Trench M., Keegan O., O’Donoghue K., Nuzum D. (2022). The Experiences of Men Following Recurrent Miscarriage in an Irish Tertiary Hospital: A Qualitative Analysis. Health Expect..

[123] Wojnar D. (2007). Miscarriage Experiences of Lesbian Couples. J. Midwifery Women’s Health.

[124] Krieg S.A., Shahine L.K., Lathi R.B. (2016). Environmental Exposure to Endocrine-Disrupting Chemicals and Miscarriage. Fertil. Steril..

[125] Darrow L.A., Howards P.P., Winquist A., Steenland K. (2014). PFOA and PFOS Serum Levels and Miscarriage Risk. Epidemiology.

[126] Li Y., Xu Y., Scott K., Lindh C., Jakobsson K., Fletcher T. (2019). Half-Lives of PFOA, PFPeS, PFHxS, PFHpS and PFOS after End of Exposure to Contaminated Drinking Water. Environ. Epidemiol..

[127] Kolte A.M., Bernardi L.A., Christiansen O.B., Quenby S., Farquharson R.G., Goddijn M., Stephenson M.D. (2014). Terminology for Pregnancy Loss prior to Viability: A Consensus Statement from the ESHRE Early Pregnancy Special Interest Group. Hum. Reprod..

[128] Ouyang F., Serum D.D.T. (2005). Age at Menarche, and Abnormal Menstrual Cycle Length. Occup. Environ. Med..

[129] Watkins D.J., Sánchez B.N., Téllez-Rojo M.M., Lee J.M., Mercado-García A., Blank-Goldenberg C., Peterson K.E., Meeker J.D. (2017). Phthalate and Bisphenol a Exposure during in Utero Windows of Susceptibility in Relation to Reproductive Hormones and Pubertal Development in Girls. Environ. Res..

[130] Gallo M.V., Ravenscroft J., Carpenter D.O., Frye C., Cook B., Schell L.M., Akwesasne Task Force on the Environment (2016). Endocrine Disrupting Chemicals and Ovulation: Is There a Relationship?. Environ. Res..

[131] Land K.L., Miller F.G., Fugate A.C., Hannon P.R. (2022). The Effects of Endocrine-Disrupting Chemicals on Ovarian- and Ovulation-Related Fertility Outcomes. Mol. Reprod. Dev..

[132] Gore A.C., Chappell V.A., Fenton S.E., Flaws J.A., Nadal A., Prins G.S., Toppari J., Zoeller R.T. (2015). EDC-2: The Endocrine Society’s Second Scientific Statement on Endocrine-Disrupting Chemicals. Endocr. Rev..

[133] Upson K., Shearston J.A., Kioumourtzoglou M.A. (2022). An Epidemiologic Review of Menstrual Blood Loss as an Excretion Route for Per- and Polyfluoroalkyl Substances. Curr. Environ. Health Rep..

[134] Wong F., MacLeod M., Mueller J.F., Cousins I.T. (2014). Enhanced Elimination of Perfluorooctane Sulfonic Acid by Menstruating Women: Evidence from Population-Based Pharmacokinetic Modeling. Environ. Sci. Technol..

[135] Ely D., Hamilton B. (2018). Trends in Fertility and Mother’s Age at First Birth Among Rural and Metropolitan Counties: United States, 2007–2017. Centers for Disease Control and Prevention: National Center for Health Statistics. https://www.cdc.gov/nchs/data/databriefs/db323-h.pdf.

[136] Lee Y.J., Jung H.W., Kim H.Y., Choi Y.-J., Lee Y.A. (2021). Early-Life Exposure to Per- and Poly-Fluorinated Alkyl Substances and Growth, Adiposity, and Puberty in Children: A Systematic Review. Front. Endocrinol..

[137] Anastasiadis X., Matsas A., Panoskaltsis T., Bakas P., Papadimitriou D.T., Christopoulos P. (2023). Impact of Chemicals on the Age of Menarche: A Literature Review. Children.

[138] Knutsen H.K., Alexander J., Barregård L., Bignami M., Brüschweiler B., Ceccatelli S., Cottrill B., Dinovi M., Edler L., Grasl-Kraupp B. (2018). Risk to Human Health Related to the Presence of Perfluorooctane Sulfonic Acid and Perfluorooctanoic Acid in Food. EFSA J..

[139] Ding N., Harlow S.D., Randolph J.F., Loch-Caruso R., Park S.K. (2020). Perfluoroalkyl and Polyfluoroalkyl Substances (PFAS) and Their Effects on the Ovary. Hum. Reprod. Update.

[140] Chang C.-J., Ryan P.B., Smarr M.M., Kannan K., Panuwet P., Dunlop A.L., Corwin E.J., Barr D.B. (2021). Serum Per- and Polyfluoroalkyl Substance (PFAS) Concentrations and Predictors of Exposure among Pregnant African American Women in the Atlanta Area, Georgia. Environ. Res..

[141] Park S.K., Peng Q., Ding N., Mukherjee B., Harlow S.D. (2019). Determinants of Per- and Polyfluoroalkyl Substances (PFAS) in Midlife Women: Evidence of Racial/Ethnic and Geographic Differences in PFAS Exposure. Environ. Res..

[142] Taylor K.W., Hoffman K., Thayer K.A., Daniels J.L. (2014). Polyfluoroalkyl Chemicals and Menopause among Women 20–65 Years of Age (NHANES). Environ. Health Perspect..

[143] Knox S.S., Jackson T., Javins B., Frisbee S.J., Shankar A., Ducatman A. (2011). Implications of Early Menopause in Women Exposed to Perfluorocarbons. J. Clin. Endocrinol. Metab..

[144] Mathews T.J., Hamilton B.E. (2009). Delayed Childbearing: More Women Are Having Their First Child Later in Life. NCHS Data Brief, no 21. Hyattsville, MD: National Center for Health Statistics. https://www.cdc.gov/nchs/data/databriefs/db21.pdf.

[145] Birth Characteristics in England and Wales-Office for National Statistics. https://www.ons.gov.uk/peoplepopulationandcommunity/birthsdeathsandmarriages/livebirths/bulletins/birthcharacteristicsinenglandandwales/2021.

[146] De Vos M., Devroey P., Fauser B.C. (2010). Primary Ovarian Insufficiency. Lancet.

[147] Webber L., Davies M., Anderson R., Bartlett J., Braat D., Cartwright B., Cifkova R., de Muinck Keizer-Schrama S., Hogervorst E., Janse F. (2016). ESHRE Guideline: Management of Women with Premature Ovarian Insufficiency. Hum. Reprod..

[148] Vabre P., Gatimel N., Moreau J., Gayrard V., Picard-Hagen N., Parinaud J., Leandri R.D. (2017). Environmental Pollutants, a Possible Etiology for Premature Ovarian Insufficiency: A Narrative Review of Animal and Human Data. Environ. Health.

[149] Gao Y., Hong X., Wang Z., Zhu Y. (2017). Endometrial Receptivity and Conception Outcome among Women with Light Menstrual Bleeding of Unidentified Etiology. Int. J. Gynecol. Obstet..

[150] Carroll J., Saxena R., Welt C.K. (2012). Environmental and Genetic Factors Influence Age at Menarche in Women with Polycystic Ovary Syndrome. J. Pediatr. Endocrinol. Metab..

[151] Cheng T.S., Day F.R., Lakshman R., Ong K.K. (2020). Association of Puberty Timing with Type 2 Diabetes: A Systematic Review and Meta-Analysis. PLoS Med..

[152] Yoo J.-H. (2016). Effects of Early Menarche on Physical and Psychosocial Health Problems in Adolescent Girls and Adult Women. Korean J. Pediatr..

[153] Schaeffer K., Aragão C. Key Facts About Moms in the U.S. Pew Research Center. https://www.pewresearch.org/short-reads/2023/05/09/facts-about-u-s-mothers/#:~:text=In%202021%2C%20the%20average%20woman.

[154] Schell L.M., West C.N. (2023). Age at Menarche and Chemical Exposure: Per- and Polyfluoroalkyl Substances (PFAS), Dichloro-Diphenyl-Trichloroethane (DDT), Dichloro-Diphenyl-Dichloroethylene (DDE), and Polychlorinated Biphenyls (PCBs). Ann. Hum. Biol..

[155] Farahmand M., Ramezani Tehrani F., Azizi F. (2012). Whether Age of Menarche Is Influenced by Body Mass Index and Lipoproteins Profile? A Retrospective Study. Iran. J. Reprod. Med..

[156] Gong T.-T., Wang Y.-L., Ma X.-X. (2015). Age at Menarche and Endometrial Cancer Risk: A Dose-Response Meta-Analysis of Prospective Studies. Sci. Rep..

[157] Bullard R.D., Mohai P., Saha R., Wright B. (2008). Toxic Wastes and Race at Twenty: Why Race Still Matters After All of These Years. Environ. Law.

[158] Mohai P., Saha R. (2015). Which Came First, People or Pollution? A Review of Theory and Evidence from Longitudinal Environmental Justice Studies. Environ. Res. Lett..

[159] Santaliz Casiano A., Lee A., Teteh D., Madak Erdogan Z., Treviño L. (2022). Endocrine-Disrupting Chemicals and Breast Cancer: Disparities in Exposure and Importance of Research Inclusivity. Endocrinology.

[160] James-Todd T.M., Chiu Y.-H., Zota A.R. (2016). Racial/Ethnic Disparities in Environmental Endocrine Disrupting Chemicals and Women’s Reproductive Health Outcomes: Epidemiological Examples across the Life Course. Curr. Epidemiol. Rep..

[161] Office of Minority Health Infant Mortality and African Americans. https://minorityhealth.hhs.gov/infant-mortality-and-african-americans#:~:text=Non%2DHispanic%20blacks%2FAfrican%20Americans.

[162] Centers for Disease Control and Prevention Infant Mortality. https://www.cdc.gov/reproductivehealth/maternalinfanthealth/infantmortality.htm.

[163] Wallace M., Crear-Perry J., Richardson L., Tarver M., Theall K. (2017). Separate and Unequal: Structural Racism and Infant Mortality in the US. Health Place.

[164] Pabayo R., Ehntholt A., Davis K., Liu S.Y., Muennig P., Cook D.M. (2019). Structural Racism and Odds for Infant Mortality among Infants Born in the United States 2010. J. Racial Ethn. Health Disparities.

[165] Chen F., Yin S., Kelly B.C., Liu W. (2017). Isomer-Specific Transplacental Transfer of Perfluoroalkyl Acids: Results from a Survey of Paired Maternal, Cord Sera, and Placentas. Environ. Sci. Technol..

[166] Rush E.L., Singer A.B., Longnecker M.P., Haug L.S., Sabaredzovic A., Symanski E., Whitworth K.W. (2018). Oral Contraceptive Use as a Determinant of Plasma Concentrations of Perfluoroalkyl Substances among Women in the Norwegian Mother and Child Cohort (MoBa) Study. Environ. Int..

[167] Faubion S.S., Kuhle C.L., Shuster L.T., Rocca W.A. (2015). Long-Term Health Consequences of Premature or Early Menopause and Considerations for Management. Climacteric.

[168] Tsiligiannis S., Panay N., Stevenson J.C. (2019). Premature Ovarian Insufficiency and Long-Term Health Consequences. Curr. Vasc. Pharmacol..

[169] Hernández-Angeles C., Castelo-Branco C. (2016). Early Menopause: A Hazard to a Woman’s Health. Indian J. Med. Res..

[170] Bozdag G., Mumusoglu S., Zengin D., Karabulut E., Yildiz B.O. (2016). The Prevalence and Phenotypic Features of Polycystic Ovary Syndrome: A Systematic Review and Meta-Analysis. Hum. Reprod..

[171] Balen A.H., Rutherford A.J. (2007). Managing Anovulatory Infertility and Polycystic Ovary Syndrome. BMJ.

[172] Bulletti C., Coccia M.E., Battistoni S., Borini A. (2010). Endometriosis and Infertility. J. Assist. Reprod. Genet..

[173] Chambers W.S., Hopkins J.G., Richards S.M. (2021). A Review of Per- and Polyfluorinated Alkyl Substance Impairment of Reproduction. Front. Toxicol..

[174] Crawford N.M., Fenton S.E., Strynar M., Hines E.P., Pritchard D.A., Steiner A.Z. (2017). Effects of Perfluorinated Chemicals on Thyroid Function, Markers of Ovarian Reserve, and Natural Fertility. Reprod. Toxicol..

[175] The Rotterdam ESHRE/ASRM-sponsored PCOS consensus workshop group (2004). Revised 2003 Consensus on Diagnostic Criteria and Long-Term Health Risks Related to Polycystic Ovary Syndrome (PCOS). Hum. Reprod..

[176] Purwar A., Nagpure S. (2022). Insulin Resistance in Polycystic Ovarian Syndrome. Cureus.

[177] Rosenfield R.L., Ehrmann D.A. (2016). The Pathogenesis of Polycystic Ovary Syndrome (PCOS): The Hypothesis of PCOS as Functional Ovarian Hyperandrogenism Revisited. Endocr. Rev..

[178] Barber T.M., Hanson P., Weickert M.O., Franks S. (2019). Obesity and Polycystic Ovary Syndrome: Implications for Pathogenesis and Novel Management Strategies. Clin. Med. Insights Reprod. Health.

[179] Sam S. (2007). Obesity and Polycystic Ovary Syndrome. Obes. Manag..

[180] Vagi S.J., Azziz-Baumgartner E., Sjödin A., Calafat A.M., Dumesic D., Gonzalez L., Kato K., Silva M.J., Ye X., Azziz R. (2014). Exploring the Potential Association between Brominated Diphenyl Ethers, Polychlorinated Biphenyls, Organochlorine Pesticides, Perfluorinated Compounds, Phthalates, and Bisphenol a in Polycystic Ovary Syndrome: A Case–Control Study. BMC Endocr. Disord..

[181] Campbell S., Raza M., Pollack A.Z. (2016). Perfluoroalkyl Substances and Endometriosis in US Women in NHANES 2003–2006. Reprod. Toxicol..

[182] Louis G.M.B., Peterson C.M., Chen Z., Hediger M.L., Croughan M.S., Sundaram R., Stanford J.B., Fujimoto V.Y., Varner M.W., Giudice L.C. (2012). Perfluorochemicals and Endometriosis. Epidemiology.

[183] Kang Q., Gao F., Zhang X., Wang L., Liu J., Fu M., Zhang S., Wan Y., Shen H., Hu J. (2020). Nontargeted Identification of Per- and Polyfluoroalkyl Substances in Human Follicular Fluid and Their Blood-Follicle Transfer. Environ. Int..

[184] Wang W., Zhou W., Wu S., Liang F., Li Y., Zhang J., Cui L., Feng Y., Wang Y. (2019). Perfluoroalkyl Substances Exposure and Risk of Polycystic Ovarian Syndrome Related Infertility in Chinese Women. Environ. Pollut..

[185] Radke E.G., Christensen K. (2023). Invited Perspective: Challenges in Evaluating the Effect of Per- and Polyfluoroalkyl Substance Mixtures on Polycystic Ovarian Syndrome. Environ. Health Perspect..

[186] Purdue M.P., Rhee J., Denic-Roberts H., McGlynn K.A., Byrne C., Sampson J.N., Botelho J.C., Calafat A.M., Rusiecki J. (2023). A Nested Case–Control Study of Serum Per- and Polyfluoroalkyl Substances and Testicular Germ Cell Tumors among U.S. Air Force Servicemen. Environ. Health Perspect..

[187] Corsini C., Boeri L., Candela L., Pozzi E., Belladelli F., Capogrosso P., Fallara G., Schifano N., Cignoli D., Ventimiglia E. (2023). Is There a Relevant Clinical Impact in Differentiating Idiopathic Versus Unexplained Male Infertility?. World J. Men’s Health.

[188] Krzastek S.C., Farhi J., Gray M., Smith R.P. (2020). Impact of Environmental Toxin Exposure on Male Fertility Potential. Transl. Androl. Urol..

[189] Selvaraju V., Baskaran S., Agarwal A., Henkel R. (2020). Environmental Contaminants and Male Infertility: Effects and Mechanisms. Andrologia.

[190] Donkin I., Barrès R. (2018). Sperm Epigenetics and Influence of Environmental Factors. Mol. Metab..

[191] Chen C.-E., Yang Y., Zhao J.-L., Liu Y.-S., Hu L.-X., Li B., Li C.-L., Ying G.-G. (2021). Legacy and Alternative Per- and Polyfluoroalkyl Substances (PFASs) in the West River and North River, South China: Occurrence, Fate, Spatio-Temporal Variations and Potential Sources. Chemosphere.

[192] Fu Y., Wang T., Wang P., Fu Q., Lu Y. (2014). Effects of Age, Gender and Region on Serum Concentrations of Perfluorinated Compounds in General Population of Henan, China. Chemosphere.

[193] Cooper T.G., Noonan E., von Eckardstein S., Auger J., Baker H.W.G., Behre H.M., Haugen T.B., Kruger T., Wang C., Mbizvo M.T. (2009). World Health Organization Reference Values for Human Semen Characteristics. Hum. Reprod. Update.

[194] World Health Organization (2010). WHO Laboratory Manual for the Examination and Processing of Human Semen.

[195] Dassuncao C., Hu X.C., Zhang X., Bossi R., Dam M., Mikkelsen B., Sunderland E.M. (2017). Temporal Shifts in Poly- and Perfluoroalkyl Substances (PFASs) in North Atlantic Pilot Whales Indicate Large Contribution of Atmospheric Precursors. Environ. Sci. Technol..

[196] Weihe P., Debes Joensen H. (2012). Dietary Recommendations Regarding Pilot Whale Meat and Blubber in the Faroe Islands. Int. J. Circumpolar Health.

[197] Weihe P., Kato K., Calafat A.M., Nielsen F., Wanigatunga A.A., Needham L.L., Grandjean P. (2008). Serum Concentrations of Polyfluoroalkyl Compounds in Faroese Whale Meat Consumers. Environ. Sci. Technol..

[198] World Health Organization (1999). Laboratory Manual for the Examination of Human Semen and Sperm-Cervical Mucus Interaction.

[199] Menkveld R., Stander F.S., Kotze T.J.V., Kruger T.F., Zyl J.A.V. (1990). The Evaluation of Morphological Characteristics of Human Spermatozoa according to Stricter Criteria. Hum. Reprod..

[200] Barker D. (1986). Infant Mortality, Childhood Nutrition, and Ischaemic Heart Disease in England and Wales. Lancet.

[201] Dzierlenga M.W., Crawford L., Longnecker M.P. (2020). Birth Weight and Perfluorooctane Sulfonic Acid: A Random-Effects Meta-Regression Analysis. Environ. Epidemiol..

[202] Steenland K., Barry V., Savitz D. (2018). Serum Perfluorooctanoic Acid and Birthweight. Epidemiology.

[203] Gampel S.B., Nomura Y. (2014). Short and Long-Term Effects of Compromised Birth Weight, Head Circumference, and Apgar Scores on Neuropsychological Development. J. Psychol. Abnorm. Child..

[204] Hassan S., Jahanfar S., Inungu J., Craig J.M. (2021). Low Birth Weight as a Predictor of Adverse Health Outcomes during Adulthood in Twins: A Systematic Review and Meta-Analysis. Syst. Rev..

[205] Nakano Y. (2020). Adult-Onset Diseases in Low Birth Weight Infants: Association with Adipose Tissue Maldevelopment. J. Atheroscler. Thromb..

[206] Hadlock F.P., Harrist R.B., Sharman R.S., Deter R.L., Park S.K. (1985). Estimation of Fetal Weight with the Use of Head, Body, and Femur Measurements—A Prospective Study. Am. J. Obstet. Gynecol..

[207] Buck Louis G.M., Grewal J., Albert P.S., Sciscione A., Wing D.A., Grobman W.A., Newman R.B., Wapner R., D’Alton M.E., Skupski D. (2015). Racial/Ethnic Standards for Fetal Growth: The NICHD Fetal Growth Studies. Am. J. Obstet. Gynecol..

[208] Buekers J., Colles A., Cornelis C., Morrens B., Govarts E., Schoeters G. (2018). Socio-Economic Status and Health: Evaluation of Human Biomonitored Chemical Exposure to Per- and Polyfluorinated Substances across Status. Int. J. Environ. Res. Public Health.

[209] DeLuca N.M., Thomas K., Mullikin A., Slover R., Stanek L.W., Pilant A.N., Cohen Hubal E.A. (2023). Geographic and Demographic Variability in Serum PFAS Concentrations for Pregnant Women in the United States. J. Expo. Sci. Environ. Epidemiol..

[210] Kim M.K., Lee S.M., Bae S.-H., Kim H.J., Lim N.G., Yoon S.-J., Lee J.Y., Jo M.-W. (2018). Socioeconomic Status Can Affect Pregnancy Outcomes and Complications, Even with a Universal Healthcare System. Int. J. Equity Health.

[211] Buck Louis G.M., Zhai S., Smarr M.M., Grewal J., Zhang C., Grantz K.L., Hinkle S.N., Sundaram R., Lee S., Honda M. (2018). Endocrine Disruptors and Neonatal Anthropometry, NICHD Fetal Growth Studies-Singletons. Environ. Int..

[212] Peterson A.K., Eckel S.P., Habre R., Yang T., Faham D., Amin M., Grubbs B.H., Farzan S.F., Kannan K., Robinson M. (2022). Detected Prenatal Perfluorooctanoic Acid (PFOA) Exposure Is Associated with Decreased Fetal Head Biometric Parameters in Participants Experiencing Higher Perceived Stress during Pregnancy in the MADRES Cohort. Environ. Adv..

[213] National Academies of Sciences, Engineering, and Medicine, Division of Behavioral and Social Sciences and Education, Health and Medicine Division, Committee on Population, Board on Health Sciences Policy, Committee on the Use of Race, Ethnicity, and Ancestry as Population Descriptors in Genomics Research (2023). Using Population Descriptors in Genetics and Genomics Research.

