# Supplementary material for: Environmental Exposure to Per- and Polyfluorylalkyl Substances (PFASs) and Reproductive Outcomes in the General Population: A Systematic Review of Epidemiological Studies
Source: Int J Environ Res Public Health. 2024 Dec 2;21(12):1615. doi: 10.3390/ijerph21121615 (PMC11675763; doi:10.3390/ijerph21121615)
Supplement: Supplementary file 1 [file ijerph-21-01615-s001.zip › ijerph-3270423-supplementary.pdf]

Newcastle-Ottawa Scale adapted for cohort and cross-sectional studies

Selection

1. Representativeness of the sample
    a. Truly representative of the average in the target population (random sampling, similar to national average) *
    b. Somewhat representative of the average in the target group (non-random sampling) *
    c. Selected group of users/convenience sample
    d. No description of the derivation of the included subjects

2. Sample size
    a. Justified and satisfactory (including sample size (power) calculation) *
    b. Not justified
    c. No information provided

3. Non-respondents
    a. Pre-specified comparability between respondents and non-respondents characteristics is established, OR the response rate is satisfactory (>80%) *
    b. Unsatisfactory response rate, unsatisfactory summary data on non-respondents
    c. No information provided

4. Ascertainment of the exposure (risk factor)
    a. Validated measurement tool **
    b. Non-validated measurement tool, but the tool is available or described *
    c. No description of measurement tool

Comparability (maximum 2 stars)

1. Comparability of subjects in different outcome groups on the basis of design or analysis. Confounding factors controlled.
    a. Study adjusts for relevant predictors/risk factors/confounders e.g. age, sex, parity, menstrual status, race/ethnicity (unless ethnically homogeneous sample) *
    b. Study adjusts for any other confounder *
    c. Study does not adjust for all relevant confounders/risk factors/information not provided

Outcome

1. Assessment of outcome (maximum 2 stars)
    a. Time-matched exposure and outcome (within 6 months)*
    b. Outcome measured by objective validated laboratory methods (such as medical record linkage) *
    c. Self-report
    d. No description/non-standard laboratory methods used

2. Statistical test
    a. Statistical test used to analyse the data clearly described, appropriate and measures of association presented (i.e., CI, mean difference, etc.) and probability level (p-value) *
    b. Statistical test not appropriate, not described or incomplete

Very low risk of bias: 9-10 points
Low risk of bias: 7-8 points
Medium risk of bias: 5-6 points
Very high risk of bias: 0-4 points

Newcastle-Ottawa Scale adapted for case-control studies

Selection

1. Is the case definition adequate?
    a. Yes, with independent validation *
    b. Yes, e.g. record linkage or self-report
    c. No description

2. Representativeness of the case
    a. Consecutive or obviously representative series of cases *
    b. Potential for selection biases or not stated

3. Selection of controls
    a. Community controls *
    b. Hospital controls
    c. No description

4. Definition of controls
    a. No history of disease (endpoint) *
    b. Hospital controls
    c. No description

5. Sample size
    a. Justified and satisfactory (including sample size (power) calculation). *
    b. Not justified
    c. No information provided

6. Non-respondents
    a. Pre-specified comparability between respondents and non-respondents characteristics is established, OR the response rate is satisfactory (>80%) *
    b. Unsatisfactory response rate, unsatisfactory summary data on non-respondents
    c. No information provided

7. Ascertainment of exposure
    a. Validated measurement tool **
    b. Non-validated measurement tool, but the tool is available or described. *
    c. No description of measurement tool

Comparability (maximum 2 stars)

1. Comparability of cases and controls on the basis of the design or analysis
    a. Study adjusts for relevant predictors/risk factors/confounders e.g. age, sex, parity, menstrual status, race/ethnicity (unless ethnically homogeneous sample) *
    b. Study adjusts for any other confounder *
    c. Study does not adjust for all relevant confounders/risk factors/information not provided.

Outcome

1. Assessment of outcome (maximum of 2 stars)
    a. Time-matched exposure and outcome (within 6 months) *
    b. Outcome measured by objective validated laboratory methods (such as medical record linkage) *
    c. Self-report
    d. No description/non-standard laboratory methods used.

Newcastle-Ottawa Scale adapted for case-control studies

2. Statistical test
    a. Statistical test used to analyse the data clearly described, appropriate and measures of association presented (i.e., CI, mean difference, etc.) and probability level (p-value) *
    b. Statistical test not appropriate, not described or incomplete.

3. Same method of ascertainment for cases and controls
    a. Yes *
    b. No

Very low risk of bias: 13-14 points
Low risk of bias: 10-12 points
Medium risk of bias: 5-9 points
Very high risk of bias: 0-4 points

Supplementary Table S1. Risk of bias scores for cohort and cross-sectional studies.

Endpoint	First author	Year	Title	Represent-ativeness of the sample	Sample size	Non-respondents	Ascertainment of exposure	Comparability of subjects	Assessment of outcome	Statistical test of outcome	Total
PTB	Bangma	2020	Identifying risk factors for levels of per- and polyfluoroalkyl substances (PFAS) in the placenta in a high-risk pregnancy cohort in North Carolina	1	0	1	2	2	2	1	9
Fertility	Björvang	2021	Persistent organic pollutants and the size of ovarian reserve in reproductive-aged women	1	0	1	2	1	1	1	7
Fertility	Björvang	2022	Follicular fluid and blood levels of persistent organic pollutants and reproductive outcomes among women undergoing assisted reproductive technologies	1	0	0	2	1	2	1	7
PTB	Chu	2020	Are perfluorooctane sulfonate alternatives safer? New insights from a birth cohort study	1	0	1	2	2	2	1	9
Fetal growth	Costa	2019	First-trimester maternal concentrations of polyfluoroalkyl substances and fetal growth throughout pregnancy	1	0	1	2	2	2	1	9
Menopause	Ding	2020	Associations of perfluoroalkyl substances with incident natural menopause the study of women's health across the nation	1	0	0	2	2	1	1	7
PTB	Eick & Hom Tepaksorn	2020	Associations between prenatal maternal exposure to per- and polyfluoroalkyl substances (PFAS) and polybrominated diphenyl ethers (PBDEs) and birth outcomes among pregnant women in San Francisco	1	0	1	2	2	1	1	8
PTB	Hall	2022	Concentrations of per- and polyfluoroalkyl substances (PFAS) in human placental tissues and associations with birth outcomes	1	0	0	2	2	2	1	8

PTB	Huo	2020	Perfluoroalkyl substances exposure in early pregnancy and preterm birth in singleton pregnancies A prospective cohort study	1	0	1	2	2	2	1	9
PTB	Kalloo	2020	Exposures to chemical mixtures during pregnancy and neonatal outcomes: The HOME study	1	0	0	2	2	2	1	8
PTB	Lauritzen	2017	Maternal serum levels of perfluoroalkyl substances and organochlorines and indices of fetal growth: a Scandinavian case-cohort study	1	0	0	2	2	2	1	8
PTB	Meng	2018	Prenatal exposure to perfluoroalkyl substances and birth outcomes:an updated analysis from the Danish National Birth Cohort	1	0	0	2	2	2	1	8
PTB	Manzano-Salgado	2017	Prenatal exposure to perfluoroalkyl substances and birth outcomes in a Spanish birth cohort	1	0	1	2	1	1	1	7
Fetal growth	Ouidir	2020	Association of maternal exposure to persistent prganic pollutants in early pregnancy with fetal growth	1	0	1	2	2	2	1	9
Sperm health	Pan	2019	Profiles of emerging and legacy per-/polyfluoroalkyl substances in matched serum and semen samples: new implications for human semen quality	1	0	0	2	2	2	1	8
Sperm health	Petersen	2018	Reproductive function in a population of young Faroese men with elevated exposure to polychlorinated biphenyls (PCBs) and perfluorinated alkylate substances (PFAS)	1	0	0	2	2	1	1	7
PTB	Sagiv	2018	Early-pregnancy plasma concentrations of perfluoroalkyl substances and birth outcomes in Project Viva: confounded by pregnancy hemodynamics?	1	0	0	2	2	1	1	7

Menstruation	Singer	2018	Menstrual cycle characteristics as determinants of plasma concentrations of perfluoroalkyl substances (PFASs) in the Norwegian Mother and Child Cohort (MoBa study)	1	0	0	2	2	1	1	7
Sperm health	Song	2018	Biomonitoring PFAAs in blood and semen samples: Investigation of a potential link between PFAAs exposure and semen mobility in China	1	0	1	2	2	2	1	9
Fertility; miscarriage	Wang	2021	Early pregnancy loss Do Per- and polyfluoroalkyl substances matter?	1	0	1	2	1	2	1	8
Fertility	Wise	2022	Correlates of plasma concentrations of per- and poly-fluoroalkyl substances among reproductive-aged Black women	1	0	1	2	2	1	1	8
Menstruation	Zhou	2017	Plasma perfluoroalkyl and polyfluoroalkyl substances concentration and menstrual cycle characteristics in preconception women	1	0	1	2	2	1	1	8

Supplementary Table S2. Risk of bias scores for case-control studies.

**Endpoint**	**First author**	**Year**	**Title**	**Case definition adequate**	**Represent -ativeness of the case**	**Selection of controls**	**Definition of controls**	**Sample size**	**Non-respon-dents**	**Ascertain-ment of exposure**	**Compara-bility**	**Assessment of outcome**	**Statistical test**	**Same method**	**Total**
Menstruation, ovarian health	Heffernan	2018	Perfluorinated alkyl acids in the serum and follicular fluid of UK women with and without polycystic ovarian syndrome undergoing fertility treatment and associations with	1	1	1	0	1	1	2	1	1	1	1	11

			hormonal and metabolic parameters												
Miscarriage	Liew	2020	Maternal plasma perfluoroalkyl substances and miscarriage: a nested case-control study in the Danish National Birth Cohort	1	1	1	1	0	1	2	2	1	1	1	12
PTB	Liu	2020	Does low maternal exposure to per- and polyfluoroalkyl substances elevate the risk of spontaneous preterm birth? A nested case–control study in China	1	1	1	1	0	0	2	2	1	1	1	11
Ovarian health	Wang	2017	Perfluoroalkyl substances and endometriosis-related infertility in Chinese women	1	1	1	1	0	1	2	2	2	1	1	13
Ovarian health	Wang	2019	Perfluoroalkyl substances exposure and risk of polycystic ovarian syndrome related infertility in Chinese women	1	1	1	1	0	0	2	2	1	1	1	11
Miscarriage	Wikström	2021	Exposure to perfluoroalkyl substances in early pregnancy and risk of sporadic first trimester miscarriage	1	1	1	1	0	1	2	1	2	1	1	12
PTB	Yang	2022	Low-level environmental per- and polyfluoroalkyl substances and	1	1	1	1	0	0	2	2	1	1	1	11

			preterm birth: A nested case-control study among a Uyghur population in northwestern China												
Menstruation	Zhang	2018	Association of perfluoroalkyl and polyfluoroalkyl substances with premature ovarian insufficiency in Chinese women	1	1	1	1	0	0	2	2	2	1	1	12
